# Identifying the spatial and temporal dynamics of molecularly-distinct glioblastoma sub-populations

**DOI:** 10.3934/mbe.2020267

**Published:** 2020-07-16

**Authors:** Bethan Morris, Lee Curtin, Andrea Hawkins-Daarud, Matthew E. Hubbard, Ruman Rahman, Stuart J. Smith, Dorothee Auer, Nhan L. Tran, Leland S. Hu, Jennifer M. Eschbacher, Kris A. Smith, Ashley Stokes, Kristin R. Swanson, Markus R. Owen

**Affiliations:** 1 School of Mathematical Sciences, University of Nottingham, Nottingham, NG7 2RD, UK; 2 Mathematical NeuroOncology Lab, Mayo Clinic, Phoenix, Arizona, 85054, USA; 3 School of Medicine and Health Sciences, University of Nottingham, Nottingham, NG7 2UH, UK; 4 Department of Cancer Biology, Mayo Clinic, Phoenix, Arizona 85054, USA; 5 Department of Radiology, Mayo Clinic, Phoenix, Arizona 85054, USA; 6 Department of Pathology, Barrow Neurological Institute - St. Joseph’s Hospital and Medical Center, Phoenix, Arizona 85013, USA; 7 Department of Neurosurgery, Barrow Neurological Institute - St. Joseph’s Hospital and Medical Center, Phoenix, Arizona 85013, USA; 8 Department of Imaging Research, Barrow Neurological Institute - St. Joseph’s Hospital and Medical Center, Phoenix, Arizona 85013, USA; 9 Department of Neurosurgery, Mayo Clinic, Phoenix, Arizona 85054, USA

**Keywords:** glioblastoma, EGFR, PDGFRA, interactions, mathematical oncology

## Abstract

Glioblastomas (GBMs) are the most aggressive primary brain tumours and have no known cure. Each individual tumour comprises multiple sub-populations of genetically-distinct cells that may respond differently to targeted therapies and may contribute to disappointing clinical trial results. Image-localized biopsy techniques allow multiple biopsies to be taken during surgery and provide information that identifies regions where particular sub-populations occur within an individual GBM, thus providing insight into their regional genetic variability. These sub-populations may also interact with one another in a competitive or cooperative manner; it is important to ascertain the nature of these interactions, as they may have implications for responses to targeted therapies.

We combine genetic information from biopsies with a mechanistic model of interacting GBM sub-populations to characterise the nature of interactions between two commonly occurring GBM sub-populations, those with EGFR and PDGFRA genes amplified. We study population levels found across image-localized biopsy data from a cohort of 25 patients and compare this to model outputs under competitive, cooperative and neutral interaction assumptions. We explore other factors affecting the observed simulated sub-populations, such as selection advantages and phylogenetic ordering of mutations, which may also contribute to the levels of EGFR and PDGFRA amplified populations observed in biopsy data.

## 1. Introduction

Glioblastomas (GBMs) are the most common type of primary brain tumour occurring in adults and are a particularly aggressive form of cancer. Standard treatment consists of surgically removing as much of the tumour as thought safe, followed by radiation and the chemotherapy temozolomide [1]. In spite of such aggressive treatment regimens, GBMs inevitably recur, with median survival times remaining at just 14.6 months [2].

A major factor that is thought to contribute to treatment failure is the heterogeneous nature of GBMs. These tumours are known to be comprised of multiple sub-populations of cancerous cells, each with different genetic and phenotypic features [3]. Further to this, the particular sub-populations present in a tumour vary both between patients as well as within individual tumours, creating a disease characterised by both inter- and intra-tumoural heterogeneity. In addition to this genetic heterogeneity, a similar heterogeneity in response to treatment is observed among patients, with some patients responding to a particular treatment well and others not so. Furthermore, treatment response variability is also observed even within some individual tumours, with responses varying from one region of the tumour to another. These heterogeneities in treatment responses are thought to be at least partly due to the underlying sub-populations present within each individual tumour and their distribution throughout the tumour region, with individual sub-populations responding to a given therapy differently. Furthermore, the presence of such genetically and phenotypically distinct sub-populations may be the cause of failure of some therapies, particularly targeted therapies, since resistant clones may pre-exist within the tumour or non-resistant cells could survive through cooperation with other tumour cells [3–6]. For this reason, gaining further understanding of this genetic heterogeneity and the implications for therapies will help to identify subgroups of patients with better responses to treatments and new targets for novel therapies.

Over recent years, advances in biopsy sampling techniques have been helping to characterize this inter- and intra-tumoural genetic heterogeneity exhibited by GBMs. Treatment decisions are based on the biomarkers present within a single biopsy specimen, which may only be representative of a small part of the tumour. This means that clinical decisions made based on this knowledge may not be the optimal therapeutic strategy to target the majority of cancerous cells composing the rest of the tumour, which may have different genetic features and respond better to another treatment option. Therefore, in order to gain more understanding of the genetic heterogeneity across the tumour region, image-localized multiple biopsy sampling techniques have been developed, allowing surgeons to collect multiple tissue samples from an individual during surgery and record information about the location in the tumour from where the sample was taken. Subsequent tissue analysis then identifies how the genetic profile of these samples differs from one region of the tumour to another, providing spatial and genetic information that gives insight into the inter- and intra-tumoural heterogeneity present in these complex tumours; examples of such techniques being employed can be found in [7–9].

While the exact mechanism by which such genetic heterogeneity arises in GBMs is currently unknown, several possible theories have been proposed to explain this. These include the theory of clonal evolution, where tumours are thought to evolve through a process of acquiring mutations and natural selection; the cancer stem cell (CSC) model, in which a small population of CSCs give rise to and maintain the tumour through self-renewal and producing phenotypically diverse daughter cells; or possibly some complementary combination of these two theories [3]. In addition to understanding how genetic heterogeneity arises in GBMs and other types of cancer, further understanding of how such heterogeneity is maintained is also needed. One suggested mechanism of heterogeneity maintenance is called interclonal cooperativity, where interactions between different tumour cell populations are thought to be important; the theory suggests that some cells may acquire mutations that result in the promotion of other tumour cell sub-populations in some way [3, 10]. One consequence of this could be that a small population of genetically-distinct tumour cells plays an important role in tumour progression and, thus, targeting such a sub-population of cells may have additional negative effects on the rest of the tumour cell population. Therefore, identifying such genetically distinct tumour cell sub-populations and understanding their interplay with other cell populations may have important implications for the success of therapies.

Two such populations of interest are those with amplification of the Epidermal Growth Factor Receptor (EGFR) and the Platelet-Derived Growth Factor Receptor Alpha (PDGFRA) genes, i.e., cells with an increased number of copies of the genes encoding each protein. While amplification status relates to the number of copies of a particular gene that a cell has in its DNA, its *copy number aberration*, it also induces overexpression of these genes in tumour cells [11]. The EGFR and PDGFRA proteins are both members of the Receptor Tyrosine Kinase (RTK) family of cell surface receptors which bind to a variety of growth factors, cytokines and hormones and play a crucial role in the regulation of the signalling that controls cell proliferation, metabolism and survival [12]. Specifically, EGFR is a receptor that, upon binding, results in the activation of pathways that lead to cell proliferation, DNA synthesis and the expression of certain oncogenes [13] and its amplification has been shown to promote invasion in GBMs [14, 15] and be an unfavourable predictor for patient survival [16]. Meanwhile, PDGFRA is a receptor that, when bound, activates signalling pathways that promote oncogenesis [17, 18]. Due to the prevalence of EGFR and PDGFRA amplified tumour cells in GBMs, occurring in 43% and 11% of GBM samples in The Cancer Genome Atlas (TCGA) database, respectively [19], these sub-populations have become prime molecular targets for therapies and a number of inhibitor drugs have been developed for this purpose [5]. These therapies targeting EGFR and PDGFRA amplified cells, however, have had limited success in GBMs in clinical trials so far [5].

Several possible mechanisms of chemoresistance to these drugs in GBMs are discussed by Nakada et al. [5]. However, one possible mechanism of chemoresistance to EGFR and PDGFRA targeted therapies of interest is through the interaction of cell sub-populations with amplification of these genes; these cells may interact in a cooperative way that facilitates their survival or, conversely, competitively, such that the targeting of one population with therapy benefits the other by removing its competitor. While the interactions between EGFR and PDGFRA amplified sub-populations are currently not well understood, it has been suggested that these cell populations may be interacting in a cooperative manner. For example, in experiments by Szerlip et al. [6] a form of cooperativity was observed between these cell populations, as combined inhibition of both receptors was needed to block activity of the PI3 kinase pathway- a pathway involved in the regulation of cell proliferation, apoptosis and migration [20]- in a mixed population of EGFR and PDGFRA amplified cells *in vitro*. In addition to this, Snuderl et al. [21] observed coexistence of these amplified sub-populations and suggested that they may co-evolve with similar fitness levels rather than compete during tumour evolution; the authors further suggest the possibility that these sub-populations cooperate to achieve a higher fitness level than each of the sub-populations individually [21, 22].

As stated previously, if EGFR and PDGFRA amplified sub-populations are indeed cooperating in GBMs, this will have implications for therapies targeting these cells and so it is important to gain more understanding of the nature of any interactions. In this paper, we present information from image-localized biopsies that provides insight into the distribution and co-occurrence of EGFR and PDGFRA amplified sub-populations throughout GBM tumours in a cohort of patients. While this provides important genetic and spatial information, this information is static and so it is difficult to extract any dynamic information that may help to identify the type of interactions occurring between these populations of tumour cells. Mathematical modelling could be a useful tool in this scenario, as models can be used to enhance current knowledge and provide deeper insight into complex biological processes. Therefore, we propose a novel mathematical model of interacting GBM sub-populations, where we investigate the effects of different interaction assumptions, namely cooperative, competitive and neutral (no) interactions, on the population level occurrence of EGFR and PDGFRA amplified cells *in silico*. We study population levels found across the image-localized biopsy data from a cohort of patients and compare this to model outputs under these different interaction assumptions. We explore additional factors affecting the patterns observed in our simulations, such as selection advantages and phylogenetic ordering of mutations, which may also contribute to the levels of EGFR and PDGFRA amplified populations observed in biopsy data. We find that competitive and neutral interactions between the amplified populations fit the amplification patterns observed across the patient cohort better than the cooperative case and that a combination of selection advantages, timings and locations at which mutations occur likely contribute to the patterns that arise in GBMs. Finally, we conduct a sensitivity analysis of our model and discuss our results and the insight they provide into the evolution of these biologically complex tumours as well as our planned future work.

## 2. Materials and methods

### 2.1. Image-localized biopsies and tissue analysis

Patients with clinically suspected GBM undergoing preoperative MRI for surgical resection were recruited and the absence of previous treatment was confirmed. Institutional review board approval was obtained, along with written and informed consent from each participant prior to enrollment. During surgery, each neurosurgeon collected an average of 4–5 tissue specimens from each tumour and typically selected targets separated by ≥ 1 cm from different regions of the tumour based on clinical feasibility (e.g., accessibility of the target site, overlying vessels, areas of the brain that directly control function). The location of each biopsy was also recorded by neurosurgeons to allow for subsequent co-registration with multiparametric magnetic resonance imaging (MRI) datasets. More detail of the biopsy collection protocol can be found in [7].

To determine whether a biopsy sample contains tumour cells with the EGFR and PDGFRA genes amplified, copy number aberration (CNA) values associated with these genes were determined for all tissue samples using array comparative genomic hybridization (aCGH) as detailed in references [7, 23, 24]. Each tissue sample was then classified as being amplified in a given gene if the corresponding CNA value was greater than a given threshold and not amplified in that gene when below or equal to that threshold. Each biopsy sample, however, is likely to contain a mixture of healthy non-cancerous cells and tumour cells without and with varying degrees of gene amplification. Thus, the CNA value will be based on a mixed signal from a sample containing a mixture of cells with potentially different numbers of copies of the genes of interest and it is unclear what an appropriate threshold should be to determine the gene amplification status, which is a topic widely discussed in the literature [11, 25].

In this work, we choose to use a CNA threshold of 2.2; this threshold is chosen based on some prior knowledge and some assumptions about the levels of EGFR and PDGFRA amplification that we expect to see in our tissue samples. Firstly, diploid cells that are not EGFR or PDGFRA gene amplified will have an associated CNA value equal to 2 [11, 25], which applies to the healthy cells and non-amplified tumour cells in the tissue samples. Secondly, we assume that the EGFR and PDGFRA amplified cell sub-populations are homogeneous with respect to their gene copy numbers and, therefore, all cells in each of these sub-populations have the same CNA value associated to each of these genes, which we choose to equal 4; this corresponds to each of the alleles in an EGFR amplified cell containing an extra copy of the EGFR gene and similarly for the PDGFRA amplified population. Finally, since the neurosurgeons collect biopsy samples from various regions of each tumour, including the invasive edge where tumour cell density is low, we choose the CNA threshold in a way that will be sensitive to such low densities of EGFR and PDGFRA amplified cell sub-populations; we choose this low density threshold to be 10% of the tissue in a sample and assume that if a biopsy sample consists of 10% or less of either amplified cell sub-population, then the signal will be too low to be detectable in the corresponding CNA value and this sample will not be classed as being amplified in this gene. Therefore, the CNA threshold of 2.2 is derived as follows:

CNA value of tissue sample=(Non-amp fraction of sample × CNA value of non-amp cells)+(Amp fraction of sample × CNA value of amp cells)=(0.9×2)+(0.1×4)=2.2

We note that tumour cells can exhibit varying degrees of gene amplification; for example, an EGFR amplified cell sub-population is likely to consist of cells containing a variety of copy numbers of the EGFR gene, with cells containing more than 100 copies in some cases [11]. This means that using a low CNA threshold to determine gene amplification status of a tissue sample in this way could classify a sample containing a very low fraction of “highly amplified” cells as being gene amplified. However, we expect such cases to be rare and choose this CNA threshold to avoid excluding samples that contain cells that are amplified to lower levels.

### 2.2. A mathematical model of interacting sub-populations in glioblastoma

Over recent years numerous different approaches have been taken to modelling the growth of GBM; such approaches include examples of multiscale, lattice-based or stochastic models, with each having a focus on capturing particular properties of these complex tumours; a recent comprehensive review of mathematical approaches to modelling GBMs is given by Alfonso et al. in [26]. The approach we follow here, however, is inspired by the “Proliferation-Invasion” (PI) model, which takes the form of the well-known Fisher-KPP equation [27–32]. The PI model is a minimal model of glioblastoma growth based on the simplified definition of cancer as “uncontrolled proliferation of cells with the ability to invade” [27]; two phenomena that GBMs are well-known to exhibit aggressively. The real power of the PI model lies in its simplicity as model parameters can be estimated from patient MRI scans, allowing patient-specific predictions to be made and inform clinical practice [29, 33–35]. For this reason, we choose to adopt a similar minimal approach to modelling the co-evolution of EGFR and PDGFRA amplified sub-populations in GBMs.

Our model, therefore, takes the form of an extended PI model to account for the evolution of three genetically-distinct sub-populations defined as tumour cells with the genes encoding for EGFR (*E*), PDGFRA (*P*) and neither (*N*) protein amplified. The model is then given by the system of reaction-diffusion equations:

(2.1) $$\frac{\partial E}{\partial t}=\nabla \cdot \left(D_{E}\left(1-\frac{P+N}{K}\right)\nabla E+D_{E}\frac{E}{K}(\nabla P+\nabla N)\right)+f_{E}(E,P,N),$$

(2.2) $$\frac{\partial P}{\partial t}=\nabla \cdot \left(D_{P}\left(1-\frac{E+N}{K}\right)\nabla P+D_{P}\frac{P}{K}(\nabla E+\nabla N)\right)+f_{P}(E,P,N),$$

(2.3) $$\frac{\partial N}{\partial t}=\nabla \cdot \left(D_{N}\left(1-\frac{E+P}{K}\right)\nabla N+D_{N}\frac{N}{K}(\nabla E+\nabla P)\right)+f_{N}(E,P,N).$$

We consider this model on a one-dimensional cartesian domain, *x* ∈ [0, *L*], with zero flux boundary conditions at *x* = 0, *L*, and the initial conditions given by,

(2.4) $$E(x,0)=0,\;P(x,0)=0\text{ and }N(x,0)=\frac{100}{\sqrt{\pi }}e^{-{\left|x-x_{N}^{*}\right|}^{2}},$$

where *E*, *P* and *N* are the concentrations of each of the tumour cell sub-populations (cells/mm^3^) and $x_{N}^{*}$ defines the centre of the initial distribution of type *N* tumour cells. Thus we initiate our simulations with no *E* or *P* cells present and a small population of type *N* cells.

Similarly to the PI model, our model essentially consists of two terms to model the evolution of each cell sub-population. The first of these models the ability of each cell type to invade, given by the first term on the right hand side of each equation. Each of these terms model the net migration of each population as a type of diffusion, where the parameters *D*_*E*_, *D*_*P*_ and *D*_*N*_ represent their diffusion coefficients (mm^2^/year). We choose to incorporate this non-linear form of diffusion in our model, since we expect the migration of tumour cell sub-populations to be affected by the presence of other tumour cells and derive these terms following the volume-filling approach of Painter and Hillen [36]. This approach is based on the assumption that only a finite number of cells, the *maximum carrying capacity* (*K*), can fill a given volume, which we assume to be the same for each of the cell populations *E*, *P* and *N*; other approaches to modelling the migration of multiple GBM tumour cell sub-populations can be found in [37] and [38]. We derive an approximate value for *K* (as in [38]) by assuming that all sub-populations have cells of the same size with a radius of 10*μ*m, yielding a volume of approximately 4.189 × 10^3^*μ*m^3^. Thus, we have a maximum carrying capacity of

(2.5) $$K=\frac{1\text{ cell}}{4.189\times 10^{3}\mu {\text{m}}^{3}}{\left(\frac{10^{3}\mu \text{m}}{1\text{mm}}\right)}^{3}=2.39\times 10^{5}\frac{\text{cells}}{{\text{mm}}^{3}}.$$

Meanwhile, the uncontrolled proliferation of each tumour cell sub-population is modelled by the terms *f*_*E*_, *f*_*P*_ and *f*_*N*_, which take the form:

(2.6) $$f_{E}(E,P,N)=\rho_{E}E\left(1+\alpha_{PE}\frac{P}{K}\right)\left(1-\frac{E+P+N}{K}\right)+m_{E},$$

(2.7) $$f_{P}(E,P,N)=\rho_{P}P\left(1+\alpha_{EP}\frac{E}{K}\right)\left(1-\frac{E+P+N}{K}\right)+m_{P},$$

(2.8) $$f_{N}(E,P,N)=\rho_{N}N\left(1-\frac{E+P+N}{K}\right).$$

Each of these expressions consists of a term to model the net proliferation of each sub-population. These proliferative terms include a joint logistic growth factor, where we assume there are plenty of other resources such as oxygen and nutrients available and the proliferation of each population is limited only by the availability of space, such that no net proliferation occurs once the carrying capacity, *K*, is reached.

The other factors in the above proliferation terms can be thought of as modified net proliferation rates, where the parameters *ρ*_*E*_, *ρ*_*P*_ and *ρ*_*N*_ represent the intrinsic net proliferation rates of each population (1/year). The parameters *α*_*EP*_ and *α*_*PE*_ measure the effect of sub-population *E* on sub-population *P* and vice versa. For example, if *α*_*EP*_ > 0 in Eq (2.7), then the presence of the EGFR amplified (EGFRamp) population, *E*, promotes proliferation of the PDGFRA amplified (PDGFRAamp) population, *P*; this could be due to secretion of a growth factor that PDGFRAamp cells are sensitive to, for example. Alternatively, if *α*_*EP*_ < 0, then the net proliferation of PDGFRAamp cells reduces as the density of the EGFRamp population increases and the PDGFRAamp cells are negatively affected. Furthermore, if *α*_*EP*_ = 0, then sub-population *E* has no effect on the net proliferation of *P*. The parameter *α*_*PE*_ is defined analogously and we note that if both *α*_*EP*_ and *α*_*PE*_ are zero, then there are no additional interactions between the two populations, only competition for space. We define the types of interactions that can occur between EGFR and PDGFRA amplified sub-populations in our model and summarise them in Table 1. However, in this paper, we primarily consider a subset of three types of interactions between EGFRamp and PDGFRAamp cells, namely neutralism, competition and cooperation.

Neither sub-population *E* or *P* is present initially in our model with the initial conditions defined by Eq (2.4). Instead, we assume that each simulated tumour begins as a small population of non-amplified tumour cells (*N*) and amplification of the EGFR and PDGFRA genes arises via mutations at later times [3], consistent with a recent phylogenetic study of glioblastomas [9]. Thus, the two sub-populations, *E* and *P*, arise in the model through the terms *m*_*E*_ and *m*_*P*_ in Eqs (2.6) and (2.7). While these mutation events lead to the creation of a single EGFR or PDGFRA amplified cell and there are likely to be many such events occurring during the growth of a GBM, we assume that each of the EGFR and PDGFRA amplified sub-populations only become established within the tumour at most once. Furthermore, since we are using a PDE model more suited to modelling events on the macroscopic scale rather than single cell events, we account for these successful mutation events by introducing a small population of the mutated cells as a distribution. This means that the mutated cell population actually started growing a small amount of time before being introduced in our model. However we assume that this will not have affected the other tumour cell populations present and they only begin interacting and competing for space and resources once the population is of a certain size. We, therefore, choose *m*_*E*_ = *m*_*E*_(*x*, *t*, *N*, *N*_*E*_) and *m*_*P*_ = *m*_*P*_(*x*, *t*, *N*, *N*_*P*_) to be of the following form,

(2.9) $$m_{E}\left(x,t,N,N_{E}\right)=\frac{100}{\sqrt{\pi }}\delta \left(t-t_{E}^{*}\left(N,N_{E}\right)\right)e^{{\left|x-x_{E}^{*}\right|}^{2}},$$

(2.10) $$m_{P}\left(x,t,N,N_{P}\right)=\frac{100}{\sqrt{\pi }}\delta \left(t-t_{P}^{*}\left(N,N_{P}\right)\right)e^{{\left|x-x_{P}^{*}\right|}^{2}},$$

where *δ*(·) is the Dirac delta function, $t_{E}^{*}\left(N,N_{E}\right)$ and $t_{P}^{*}\left(N,N_{P}\right)$ are the times at which populations of EGFR and PDGFRA amplified cells, *E* and *P*, are introduced as Gaussian distributions centred at $x_{E}^{*}$ and $x_{P}^{*}$, respectively. The introduction times, $t_{E}^{*}\left(N,N_{E}\right)$ and $t_{P}^{*}\left(N,N_{P}\right)$, are defined as

(2.11) $$t_{E}^{*}\left(N,N_{E}\right)=\text{inf }\left\{t>0|\int_{0}^{L}N(x,t)\text{d}x=N_{E}\right\},$$

(2.12) $$t_{P}^{*}\left(N,N_{P}\right)=\text{inf }\left\{t>0|\int_{0}^{L}N(x,t)\text{d}x=N_{P}\right\},$$

so that each sub-population is introduced when the non-amplified tumour population (*N*(*x*, *t*)) has grown to a chosen total size, *N*_*E*_ or *N*_*P*_ (measured in cells/mm^2^). Our choices for *N*_*E*_ or *N*_*P*_ are discussed further in Section 3.3. Although we have used Gaussian distributions, the specific form of distribution does not significantly change our model simulations, as long as they were chosen consistently, due to the smoothing effects of diffusion in the model.

Before moving on to presenting the results of this paper, we observe briefly that under the assumption that the EGFR and PDGFRA amplified sub-populations do not interact with one another, i.e., *α*_*EP*_ = *α*_*PE*_ = 0, and all three sub-populations diffuse and proliferate at the same rates, i.e., *D*_*E*_ = *D*_*P*_ = *D*_*N*_ and *ρ*_*E*_ = *ρ*_*P*_ = *ρ*_*N*_, then the system can be reduced to a single equation governing the total population of tumour cells, *T* = *E* + *P* + *N*, for all $t\neq \left\{t_{E}^{*},t_{P}^{*}\right\}$; this equation is the well-known, clinically significant Proliferation-Invasion (PI) model that describes the evolution of a single homogeneous population of GBM tumour cells mentioned at the start of this section. Therefore, we note that our model is consistent with the PI model when used to model a single homogeneous population *T*.

For the remainder of this paper, we consider a domain length of *L* = 200 mm. Since we are only interested in running simulations to a biologically relevant size (discussed further in Section 3.3), and all populations are introduced close to its centre, this domain is sufficiently large that tumour growth remains far from the boundaries, hence avoiding boundary condition artefacts. All simulations are produced using MatLab R2017a to implement a finite difference scheme in space (uniform mesh size of 0.25 mm) and a Forward Euler time step of 1/1500 years.

## 3. Results

### 3.1. Our model predicts that distinct competing sub-populations of tumour cells can coexist in the same tumour region

**Figure 1.**
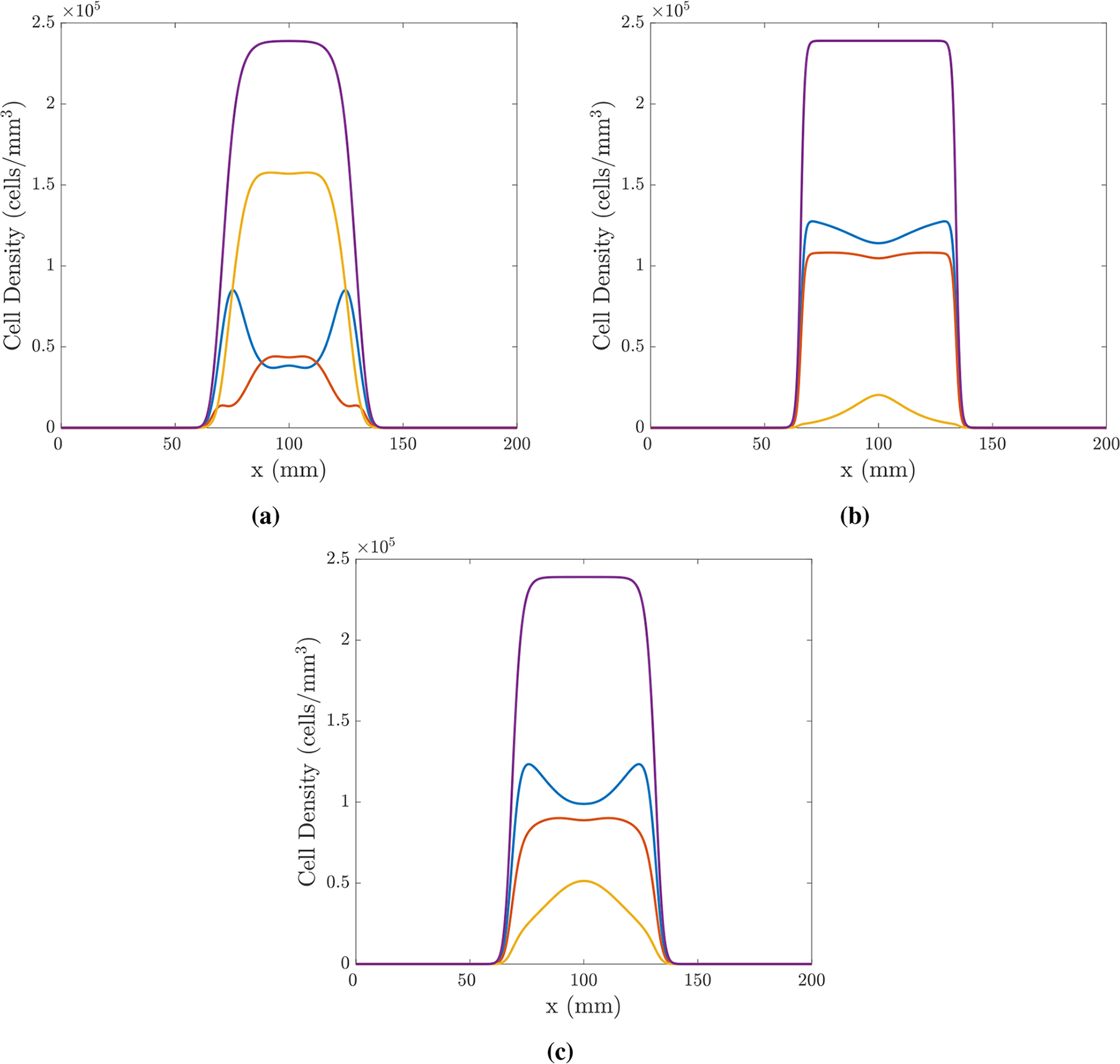
Simulations in 1D of the model given by Eqs (2.1)–(2.12) using the finite difference scheme described in Section 2.2 with parameters: *ρ*_*E*_ = 35.4, *ρ*_*P*_ = 33 and *ρ*_*N*_ = 30/year; *D*_*E*_ = *D*_*P*_ = *D*_*N*_ = 30mm^2^/year; $K_{E}^{*}=K_{P}^{*}=K_{N}^{*}=K=2.39\times 10^{5}$ cells/mm^3^; $x_{E}^{*}=x_{P}^{*}=x_{N}^{*}=100\text{mm}$; $t_{E}^{*}=0.027$, $t_{P}^{*}=0.001$ years. The interactions in each simulation are chosen to be (a) competition, *α*_*EP*_ = *α*_*PE*_ = −5, (b) cooperation, *α*_*EP*_ = *α*_*PE*_ = 5 and (c) neutralism, *α*_*EP*_ = *α*_*PE*_ = 0. Each simulation is plotted at *t* = 0.7 years; population *E* is shown in blue, *P* in red, *N* in yellow and the total population, *T* = *E* + *P* + *N*, in purple.

Intuitively, we expect that cell populations actively competing with one another may be less likely to coexist within the same region of a tumour and that their coexistence may indicate a cooperative relationship, as Snuderl et al. [21] suggest after finding intermingled sub-populations of EGFR and PDGFRA amplified sub-populations in a small number of GBM samples. In our model, however, we find that EGFR and PDGFRA amplified sub-populations can be found to coexist in areas of the tumour region despite actively competing with one another; we find that any $\bar{E}$, $\bar{P}$, $\bar{N}\geq 0$ satisfying $\bar{E}+\bar{P}+\bar{N}=K$ is a spatially homogeneous steady state and can be connected to other spatially homogeneous steady states satisfying the same condition, or the trivial steady state $\bar{E}=\bar{P}=\bar{N}=0$, by travelling wave-like solutions expanding outwards from the origin of the tumour. Indeed, such co-occurrence of EGFR and PDGFRA amplified cell sub-populations can be observed with competitive, cooperative or neutral interactions; an example of simulations with different *α*_*EP*_ and *α*_*PE*_ values to represent each of these interaction types is shown in Figure 1, where such co-occurrence is observed.

While this demonstrates that the coexistence of EGFR and PDGFRA amplified cell populations in one known region of a tumour can occur when they are competing, cooperating and evolving neutrally, it highlights the need for more information to determine the nature of such interactions between co-occurring cells *in vivo*. In this work we hope to shed some light on this by studying amplification patterns observed across image-localized biopsies from a cohort of patients and the patterns that emerge in simulations of our model, given by Eqs (2.1)–(2.12), under different interaction assumptions.

### 3.2. Biopsy data

**Figure 2.**
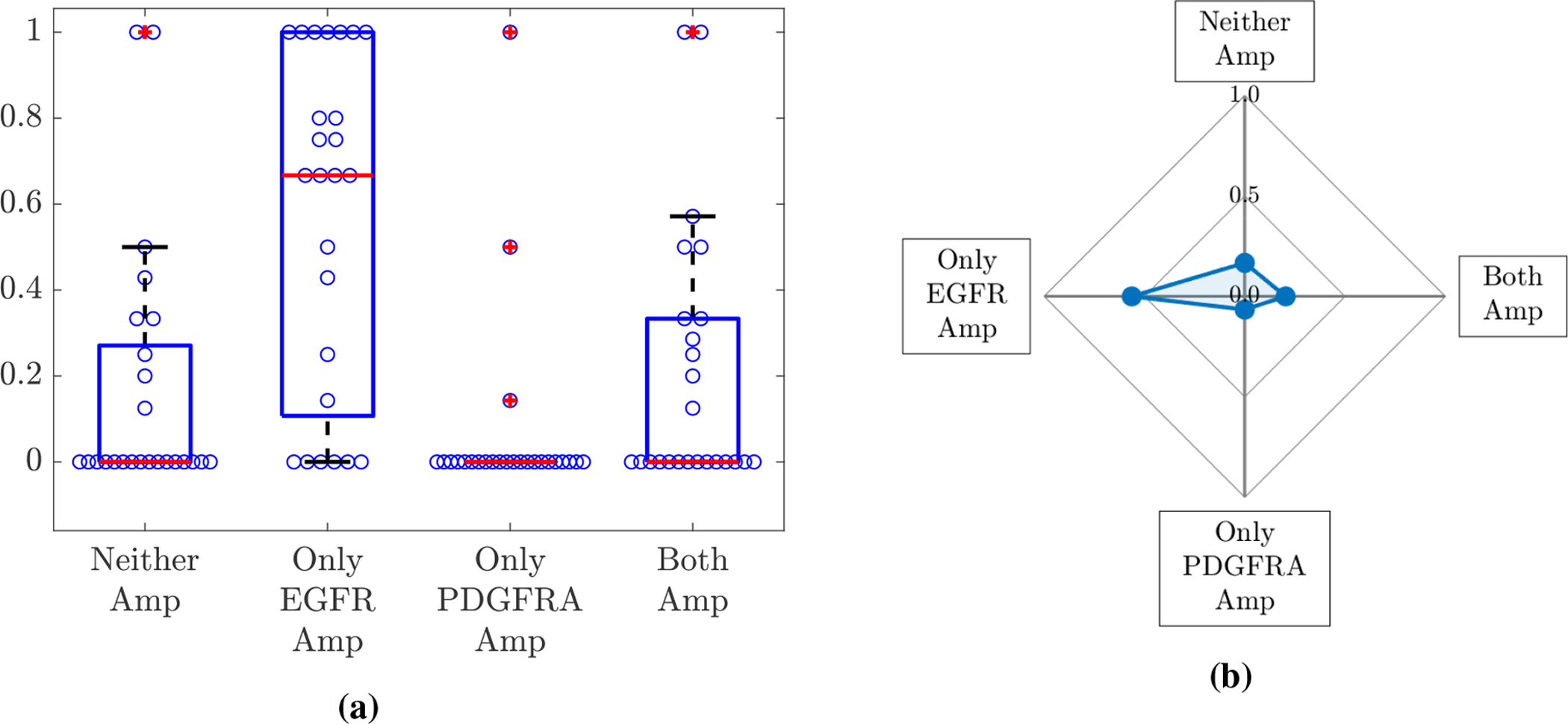
(a) A box plot summarising the proportion of each individual’s biopsies that were determined to be amplified in neither gene (Neither), only the EGFR gene (Only EGFR Amp), only the PDGFRA gene (Only PDGFRA Amp) and both of the EGFR and PDGFRA genes (Both Amp) for the 25 patients. Each of the blue circles overlaid on the box plot represents the relevant proportion of an individual’s biopsies for each category. The means of these proportions across the 25 patients are shown in (b).

A total of 120 biopsies were collected from 25 patients with clinically suspected GBM, with 2–14 collected from each individual. Of these biopsies, 95 samples from 25 patients contained adequate tumour and/or DNA content for EGFR and PDGFRA amplification status to be determined successfully through aCGH analysis. EGFR amplification was the more commonly observed genetic alteration, with 73/95 samples having a CNA value associated with EGFR amplification, whereas 28/95 were determined to be PDGFRA amplified. Of these amplified samples, 22 were found to have amplification of both the EGFR and PDGFRA genes. For each patient, we then determined the proportion of their biopsies that were found to be amplified in neither gene, only the EGFR gene, only the PDGFRA gene and, finally, both of the EGFR and PDGFRA genes simultaneously. The proportions calculated for each of the 25 patients are summarised as a box plot in Figure 2a and the mean of these proportions are shown as a spider plot in Figure 2b.

### 3.3. Simulation results

We begin by assuming that amplification of the EGFR or PDGFRA gene does not result in either sub-population, *E* or *P* in the model, acquiring any selective advantages over non-amplified cells. In other words, we choose all proliferation and invasion parameters to be the same for each population, i.e., *ρ*_*E*_ = *ρ*_*P*_ = *ρ*_*N*_ = *ρ* and *D*_*E*_ = *D*_*P*_ = *D*_*N*_ = *D*. Since the biopsy data (Figure 2b) is the mean of a cohort containing 25 individual tumours, which will vary in their proliferative and invasive potential (quantified by *ρ* and *ρ*/*D* in the PI model [33]), we use a variety of *ρ* and *D* pairs to reflect the heterogeneity seen in the patient cohort. We therefore produce four simulations using two values for each of *ρ* and *D* to mirror the range of parameters observed in unpublished patient databases and assume this cohort are similarly distributed; to represent high parameter values we use *ρ* = 30/year and *D* = 30mm^2^/year and for low values we use *ρ* = 3/year and *D* = 3mm^2^/year as in [40]. We also assume that any interactions between the EGFR and PDGFRA amplified cells affect each sub-population to the same degree, i.e., *α*_*EP*_ = *α*_*PE*_ = *α*. To represent competition and cooperation, we simulate with *α* = −5 and 5, respectively, while for neutralism *α* = 0. For now, we assume that all populations, *E*, *P* and *N*, are introduced at the centre of the domain $\left(x_{E}^{*}=x_{P}^{*}=x_{N}^{*}=100\text{mm}\right)$ and that the mutation events for the introduction of EGFR and PDGFRA amplified sub-populations occur at the same time ($t_{E}^{*}=t_{P}^{*}$, i.e., *N*_*E*_ = *N*_*P*_). Recall that the introduction times depend on the size of the non-amplified tumour population (*N*), through Eqs (2.11) and (2.12). Since the time to reach a specific tumour size depends on *ρ*, rather than introduce these populations at a specified point in time, we introduce them after a given number of proliferation events have occurred, i.e., a specified stage in the evolution of the tumours. In this case we introduce the amplified populations after the tumour has grown to six times its initial size, that is *N*_*E*_ = *N*_*P*_ = 6*N*_*I*_, where $N_{I}=\int_{0}^{L}N(x,0)\text{d}x$. While this may seem particularly early in the evolution of our simulated tumours, it is necessary to be able to investigate the amplification patterns we observe under different interactions in our model simulations; if we introduce the mutated populations later, e.g., when the *N* population has grown to 11*N*_*I*_, we do not see them growing to detectable levels in our simulations.

In order to test whether neutral, competitive or cooperative interactions between EGFR and PDGFRA amplified sub-populations best describe the patterns observed in the biopsy data, we run numerical simulations of the model described in Section 2.2 to a biologically relevant size and compare the outputs to the mean proportions of biopsies amplified in neither gene, only the EGFR gene, only the PDGFRA gene and both of the EGFR and PDGFRA genes shown in Figure 2b.

We choose a biologically relevant size to represent a typical size of a GBM tumour at the time of diagnosis, shortly after which patients will usually undergo surgery to remove as much of the tumour as feasible, with biopsy samples being collected at this time. One way to measure the typical size of a GBM at diagnosis is to segment the tumour volume visible on a T1-weighted MRI with gadolinium contrast (T1Gd MRI), a type of scan that shows the most dense area of the tumour and is typically used in the process of diagnosing a patient with a GBM. From this, the diameter of the volume-equivalent sphere can then be computed and used as a measure to indicate the size of the tumour lesion, with the average diameter at diagnosis being 36.2 mm in unpublished data. Following Swanson et al. [29], we relate the tumour volume visible on a T1Gd MRI to the volume of tumour that has a tumour cell density greater than 80% of the carrying capacity. Therefore, we choose to run simulations until the width of the total tumour cell population, *T* = *E* + *P*+ *N*, above the 0.8*K* threshold is 36.2 mm and use this as a proxy for the size of a tumour at the time of diagnosis.

**Figure 3.**
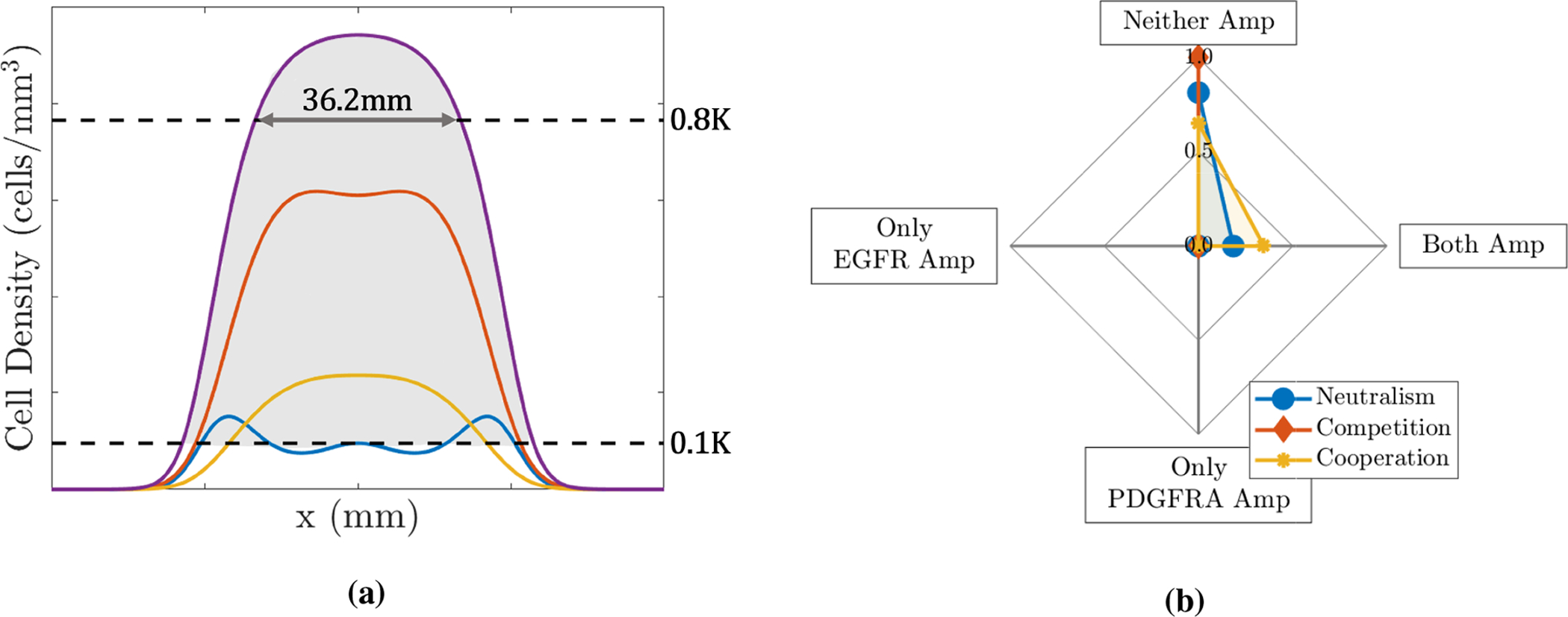
(a) Schematic illustrating the biologically relevant size that tumours are simulated to and area (shaded in grey) indicating the points with the total tumour cell population (purple curve) above the threshold of 10% of the carrying capacity. Other curves represent the individual tumour cell populations; *E* (blue), *P* (red) and *N* (yellow). (b) Plot showing the mean proportions of simulations with neither gene (Neither Amp), only the EGFR gene (Only EGFR Amp), only the PDGFRA gene (Only PDGFRA Amp) and both genes (Both Amp) amplified under different interactions when we assume that the sub-populations are dynamically the same, i.e., all populations have the same proliferation and invasion parameters, *ρ* = *ρ*_*E*_ = *ρ*_*P*_ = *ρ*_*N*_ and *D* = *D*_*E*_ = *D*_*P*_ = *D*_*N*_, and *E* and *P* populations are introduced at the same position and time, $x_{E}^{*}=x_{P}^{*}=x_{N}^{*}=100\text{mm}$ and *N*_*E*_ = *N*_*P*_ = 6*N*_*I*_, as described in Section 3.3.

We then run the numerical simulations until they reach this biologically relevant size with the parameter sets described above. Since the patient data is for the proportions of biopsies containing EGFR and PDGFRA amplified cells above a given density threshold, which we chose to be 10% of the tissue sample, we define an equivalent measure for each simulated tumour by integrating solutions across the whole tissue domain. For example, the proportion of the tumour with only EGFR amplified, *A*_*E*_, is calculated as

(3.1) $$A_{E}(t)=\frac{\int_{0}^{L}H(E(x,t)-0.1K)H(0.1K-P(x,t))\text{d}x}{\int_{0}^{L}H(E(x,t)+P(x,t)+N(x,t)-0.1K)\text{d}x},$$

(3.2) $$\approx \frac{\text{Number of mesh points with }E>0.1K\text{ and }P<0.1K}{\text{Number of mesh points with }T>0.1K},$$

where *H*(·) is the Heaviside step function. The proportions with neither gene, only the PDGFRA gene and both genes amplified, *A*_*N*_(*t*), *A*_*P*_(*t*) and *A*_*B*_(*t*), are defined and calculated in a similar way. We illustrate this schematically in Figure 3a.

The mean proportions from the simulations run using each of the four *ρ* and *D* pairs with the different cases of competition, cooperation and neutralism are shown in Figure 3b. From this plot, we see that all simulations are classed as neither gene amplified in the competitive case and this proportion decreases as we move through the neutralism case to the cooperative case and the proportion with both genes amplified increases, which is as we would intuitively expect to see. We notice that in all three cases the proportions of simulations with only one of the genes amplified are zero; again, this is expected as populations *E* and *P* have the same proliferation and invasion parameters (*ρ* and *D*) and are both introduced at the same time and place and, thus, are effectively the same so we expect to see them together (or not at all in the competitive case).

Clearly, the patterns of neither, only EGFR, only PDGFRA and both amplified proportions we see in these simulations do not reflect the patterns of amplification we see in the biopsy data in Figure 2b; one obvious difference is the proportion of only EGFR amplified biopsies, which is above 0.5 in the data and is 0 in the simulations shown in Figure 3b. Since EGFR and PDGFRA amplified cells are not exclusively found in the same biopsies in the patient data and the proportions of biopsies with only one gene amplified also differ, this indicates the two populations must differ in their dynamics in some way. There are several possible ways that differences between the EGFR and PDGFRA amplified sub-populations can occur in our model: by giving them a selective advantage (changing *ρ*_*E*_, *ρ*_*P*_, *D*_*E*_ or *D*_*P*_); by changing the phylogenetic ordering of mutations (changing $t_{E}^{*}$ or $t_{P}^{*}$); by changing the location that mutations arise in the evolving tumour (changing $x_{E}^{*}$ or $x_{P}^{*}$); by changing the strength of interaction felt by each population (changing *α*_*EP*_ or *α*_*PE*_); or, finally, by changing any combination of these factors.

#### 3.3.1. Selection advantages

The amplification of EGFR and PDGFRA genes are often considered to be among the key mutations driving oncogenesis and tumour growth [21, 22]. Since these genes are both members of the RTK family of cell surface receptors that play an important role in the regulation of cell proliferation, metabolism and survival [12] and tumours identified to be EGFR amplified have shown to be more invasive [6, 15], it is reasonable to consider that amplification of these genes may drive the growth of tumours through increasing the intrinsic proliferative and invasive abilities of these cell sub-populations. We can explore the effects of this by giving the EGFR and PDGFRA amplified sub-populations in our model different selection advantages through changing the appropriate parameters, namely *ρ*_*E*_, *ρ*_*P*_, *D*_*E*_ and *D*_*P*_.

**Figure 4.**
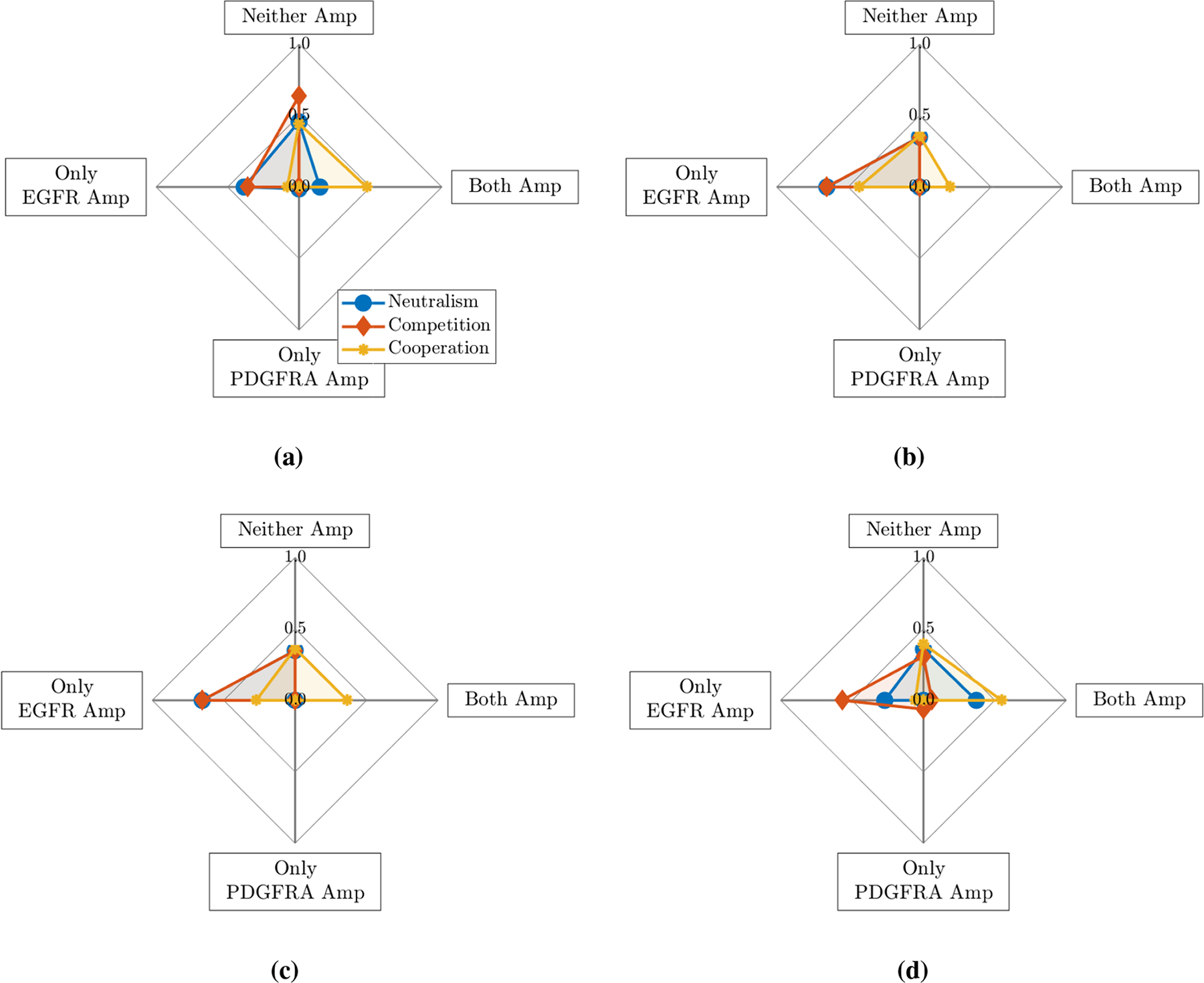
Plot showing the mean proportions of simulations with neither gene (Neither Amp), only the EGFR gene (Only EGFR Amp), only the PDGFRA gene (Only PDGFRA Amp) and both genes (Both Amp) amplified under different interactions when the *E* and *P* sub-populations are given various selection advantages: (a) EGFR 50% invasive advantage (*D*_*E*_ = 1.5*D*_*N*_, *D*_*P*_ = *D*_*N*_); (b) EGFR 50% proliferative and invasive advantage (*ρ*_*E*_ = 1.5*ρ*_*N*_, *ρ*_*P*_ = *ρ*_*N*_, *D*_*E*_ = 1.5*D*_*N*_ and *D*_*P*_ = *D*_*N*_); (c) EGFR 50% proliferative and invasive advantage, PDGFRA 50% invasive advantage (*ρ*_*E*_ = 1.5*ρ*_*N*_, *ρ*_*P*_ = *ρ*_*N*_, *D*_*E*_ = 1.5*D*_*N*_ and *D*_*P*_ = 1.5*D*_*N*_); (d) EGFR 50% proliferative and invasive advantage, PDGFRA 50% proliferative advantage (*ρ*_*E*_ = 1.5*ρ*_*N*_, *ρ*_*P*_ = 1.5*ρ*_*N*_, *D*_*E*_ = 1.5*D*_*N*_ and *D*_*P*_ = *D*_*N*_)

As we see a much higher proportion of biopsies with only EGFR amplified in the patient data (Figure 2b), this could indicate that EGFR amplified cells have a selective advantage over the PDGFRA amplified cells and those with neither gene amplified. Thus, we explore the effects of giving EGFR amplified cells invasive and proliferative advantages; in Figure 4, plots are produced from simulation results in the same way as previously described, but with (a) a 50% invasive advantage for the EGFR amplified cells and (b) additionally with a proliferative advantage. In both of these cases, there are now large proportions of simulations with EGFR amplified and without PDGFRA amplified cells present, particularly in the competitive and neutral cases. In both competitive cases in Figures 4a and 4b, we observe nowhere where both of the EGFR and PDGFRA genes are found to be amplified, unlike the patient data in Figure 2b where this was approximately 20% of biopsies. Meanwhile, the corresponding cooperative cases both gave the highest level of points with both genes amplified and the lowest levels with only EGFR amplified. In Figure 4, we also explored the effects of affording the PDGFRA amplified sub-population a (c) 50% invasive and (d) 50% proliferative advantage over non-amplified cells while giving EGFR amplified cells the same advantages as in (b). These resulted in qualitatively similar amplification patterns to those observed in (b) and (a), respectively, with the exception of the competitive case in (d) where a small proportion of simulations with only PDGFRA amplified were observed. Further plots exploring the effects of selection advantages can be found in the supplementary material in Figure 8 and are not presented here for brevity. We find that giving either, or both, of the amplified populations invasive and proliferative advantages over non-amplified cells improves the amplification patterns we see in simulations with respect to the biopsy data and, in general, the competition and neutral cases fit the biopsy data better than the cooperative case, which generally results in higher proportions of tumours with both genes amplified. Next we move on to explore the effects that the timing of mutations have on the results we see.

#### 3.3.2. The phylogenetic ordering of mutations

In all simulations presented up until this point, we have assumed that the mutations leading to the establishment of EGFR and PDGFRA amplified cell sub-populations occur at the same time. While this could be the case, a study reconstructing the phylogeny of GBMs identified EGFR and PDGFRA amplification as early and late events during tumour progression [9]. From an analysis of multiple spatially distinct samples from 11 GBMs, it was inferred that alterations that were more common occurred earlier in the evolution of the tumour compared to those only present in a smaller subset of cells. Alterations in gene copy numbers on the chromosomes where the EGFR and PDGFRA genes are found tended to occur in the early and middle phases of tumour growth, respectively [9]. Therefore, this timing, or *phylogenetic ordering*, of mutations may be affecting the proportions of EGFR and PDGFRA amplified biopsies across the patient cohort (Figure 2b) and so we undertake a brief exploration of the effect it has in our simulations.

Therefore, we assume once more that all cell sub-populations have the same proliferation and invasion parameters, i.e., *ρ* = *ρ*_*E*_ = *ρ*_*P*_ = *ρ*_*N*_ and *D* = *D*_*E*_ = *D*_*P*_ = *D*_*N*_ and, as described before, we simulate using four different *ρ* and *D* pairs and the same assumptions described in Section 3.3, with the exception of changing the times that the EGFR (*E*) and PDGFRA (*P*) amplified populations are introduced, i.e., $t_{E}^{*}$ and $t_{P}^{*}$. In the previous simulations, both populations were introduced after the non-amplified population (*N*) had grown to six times its initial size, 6*N*_*I*_, and so we now choose to investigate the patterns of gene amplification we see when the *E* and *P* populations are introduced earlier and later than this. Thus, we define a vector of possible introduction times, $t^{*}=\left(t_{1}^{*},\ldots ,t_{7}^{*}\right)$ as follows: $t_{i}^{*}$ is defined as the first time point in our simulations after the growing *N* population has reached a size of (*i* + 2)*N*_*I*_, for *i* = 1, …, 7. Implementing our model with $t_{E}^{*}$ and $t_{P}^{*}$ taking each of these values, we gain some insight into the effect that changing the time of mutations has on our simulations.

**Figure 5.**
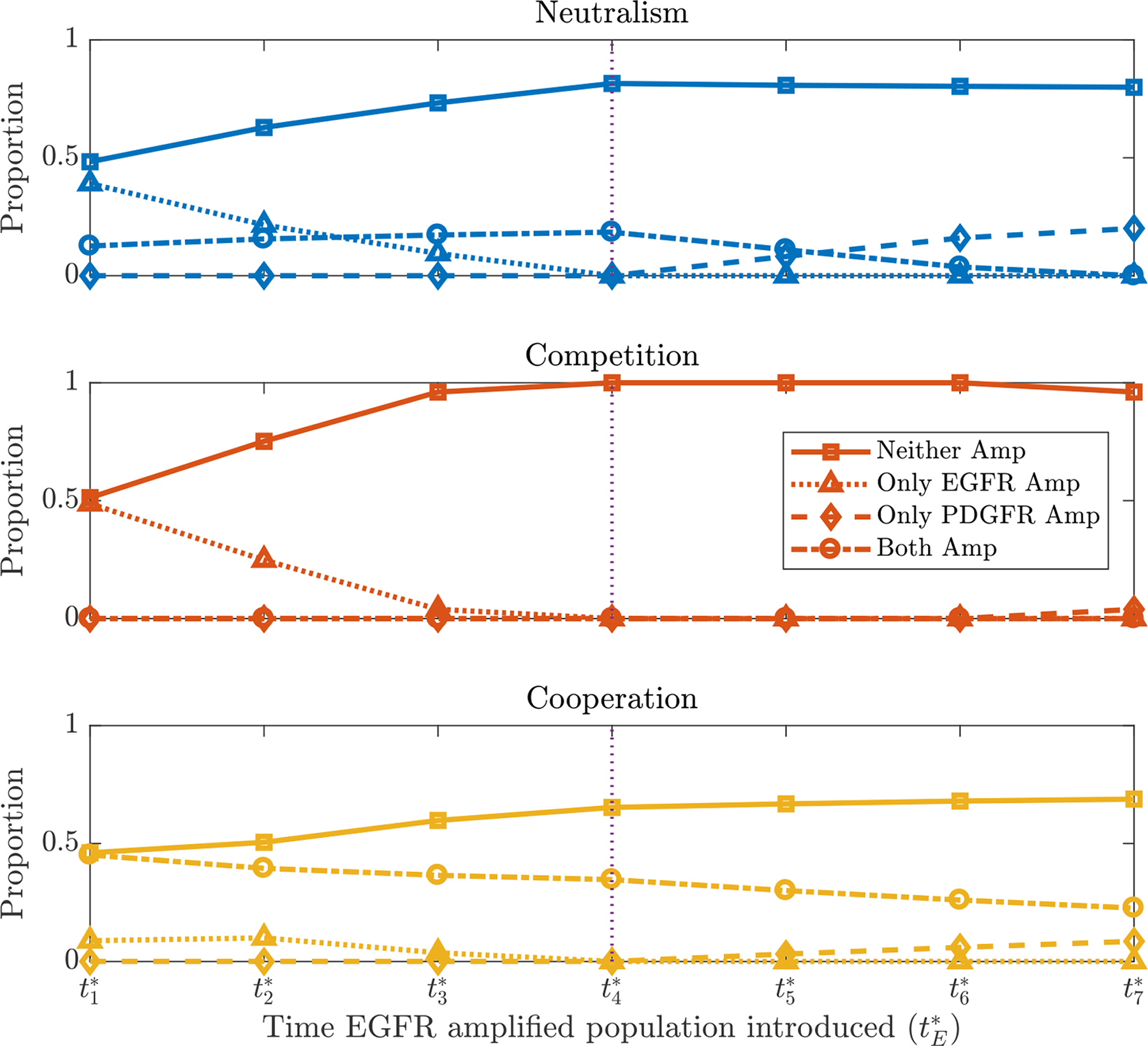
Plots showing the mean proportions of simulations with neither gene (Neither Amp), only the EGFR gene (Only EGFR Amp), only the PDGFRA gene (Only PDGFRA Amp) and both genes (Both Amp) amplified as $t_{E}^{*}$ is varied, under neutral, competitive and cooperative interactions. The PDGFRA amplified population is introduced at the fixed time $t_{P}^{*}=t_{4}^{*}$ (denoted by the vertical dotted line) and other parameters and assumptions are as described in Section 3.3.

Figure 5 shows the average amplified proportions in seven sets of simulations under neutral, competitive and cooperative interaction assumptions, where cells of type *P* are introduced at $t_{P}^{*}=t_{4}^{*}$ in each set, but the *E* population is introduced at each of the seven possible times given by the vector t*. The points on the graph where $t_{E}^{*}=t_{4}^{*}$ (marked by the dotted vertical line) are the same data represented in Figure 3b as the EGFR and PDGFRA amplified populations are introduced at the same time. From this graph, we see that the proportion with neither gene amplified decreases and that with EGFR amplified increases as the EGFR amplified population is introduced earlier. Meanwhile, as *E* cells are introduced later than *P* cells, we see the proportions changing as we would expect, with the only PDGFRA amplified proportion increasing. We also note the slight decrease in the proportion of neither amplified cells when the EGFR amplified population is introduced at $t_{E}^{*}=t_{7}^{*}$ in the competitive case, this is because the PDGFRA population is allowed enough time to proliferate to a size where the introduction and competitive interactions of EGFR amplified cells has a smaller relative effect on their proliferation. Again, for brevity we do not present all the simulation results here, further plots can be found in the supplementary material (Figures 9 and 10). As with the previous section where we looked at the effect of giving the amplified sub-populations selection advantages, we find that changing the timing of introducing the mutated populations in our simulations does not fit the biopsy data perfectly, but has some of the desired effects. For example, to get proportions more similar to the biopsy data, we need the proportion with neither gene amplified to decrease and the EGFR amplified proportion to increase, which is consistent with earlier EGFR introduction times in the competitive and neutral scenarios, whereas the cooperative case produces a much higher proportion of both amplified.

#### 3.3.3. The location of mutations

Another factor that could result in some biopsies having only EGFR and others only PDGFRA amplified is that the mutations occurred in different places, resulting in the populations occupying different, spatially separated regions of the tumour. In all previous simulations presented in this paper we assumed that the mutation events leading to the establishment of EGFR and PDGFRA amplified sup-populations in our model occurred in the center of the growing tumour; this was a reasonable place to explore the effects of selection advantages and timing of mutations from, since it is where most proliferation is taking place in our model in the early phases of tumour growth and, therefore, where we may expect more mutations to appear. However, cells at the centre of the tumour also experience more competition for space, a growth limiting resource in our model, as this is where the highest tumour cell density is in these early growth phases. Therefore, we now explore the effects of introducing the EGFR and PDGFRA amplified sub-populations away from the centre of the tumour where there is less competition for space.

**Figure 6.**
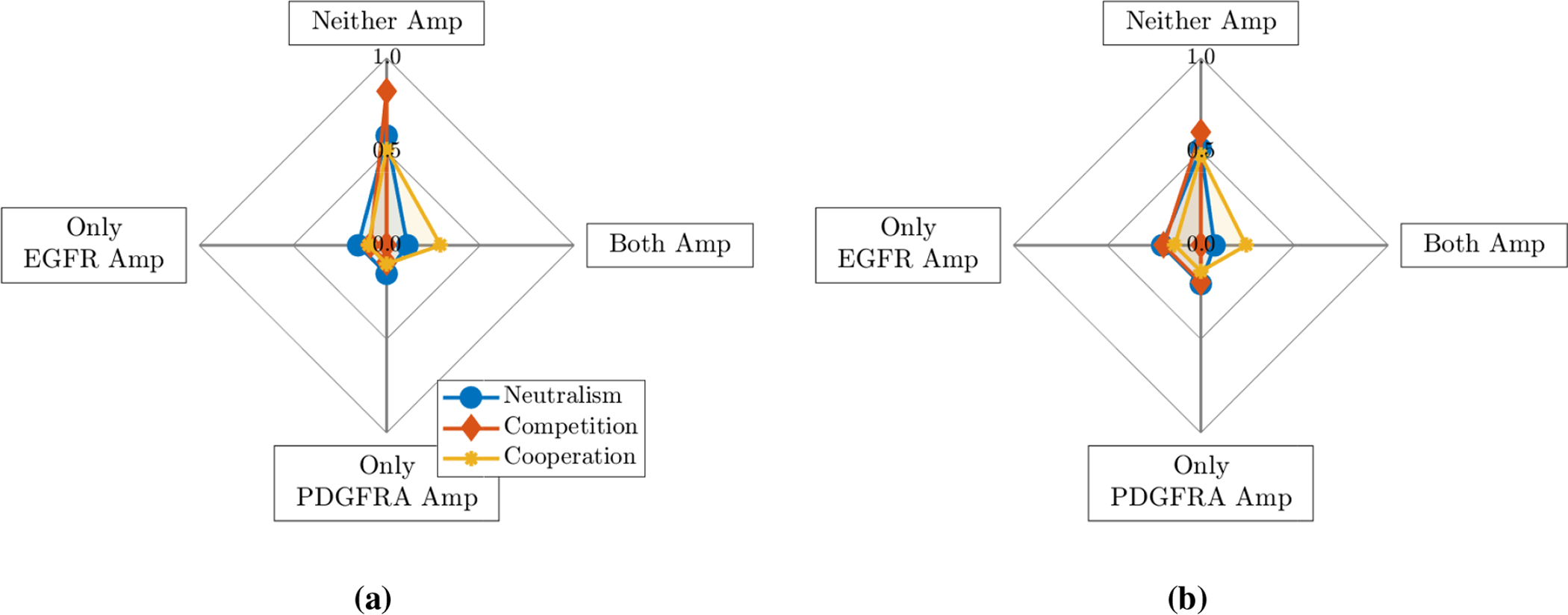
Sub-population distributions when mutations are introduced on opposite sides of the tumour: plots showing mean proportions of simulations with neither gene (Neither Amp), only the EGFR gene (Only EGFR Amp), only the PDGFRA gene (Only PDGFRA Amp) and both genes (Both Amp) amplified when EGFR and PDGFRA amplified sub-populations are introduced (a) 0.5mm to left and right of the center (i.e., $x_{E}^{*}=x_{3}^{*}$ and $x_{P}^{*}=x_{5}^{*}$) and (b) 1mm to left and right of the center (i.e., $x_{E}^{*}=x_{2}^{*}$ and $x_{P}^{*}=x_{6}^{*}$), respectively.

We use the same parameters and assumptions described in Section 3.3, but define a vector of possible introduction locations. In these simulations, the mutated populations are introduced when the growing tumour is small (the *N* population is only six times its initial size), with the bulk of the tumour being contained within a width of 3mm for each (*ρ*, *D*) pair at this time. Thus we choose introduction locations no further than 1.5mm from the tumour centre as mutations are unlikely to occur beyond this point where concentrations of tumour cells are very low and, consequently, few proliferation events are occurring. We define a vector of possible introduction locations, $x^{*}=\left(x_{1}^{*},\ldots ,x_{7}^{*}\right)$ as follows: $x_{i}^{*}=x_{c}^{*}+0.5(i-4)\text{mm}$, where $x_{c}^{*}$ is the mesh point at the centre of the tumour. We produce simulations with $x_{E}^{*}$ and $x_{P}^{*}$ each taking the values given by the vector x*. We show two simulation results in Figure 6 showing the proportions when EGFR and PDGFRA amplified populations are introduced (a) 0.5 mm and (b) 1 mm to the left and right of the tumour center, respectively. In both of these cases we see distinct regions with EGFR and PDGFRA amplification forming in the simulated tumours of equal proportion. We refer the reader to Figures 11 and 12 in the supplementary material for further results where we find that introducing each population closer to the tumour centre has a small effect on the amplified tumour proportion and that introducing PDGFRA further away from the EGFR amplified population has similar effects to those presented here in Figure 6. As found in the previous two sections, only changing where the amplified sub-populations are introduced does not fully explain the patterns of amplification we observe in the biopsy data, however it does produce some of the desired effects, such as increasing the proportion of simulations with only one of each gene amplified.

### 3.4. LHS-PRCC Sensitivity Analysis

To consider the implications of our model in a more comprehensive manner, in this section we conduct a sensitivity analysis through Latin Hyper-cube Sampling (LHS) and Partial Rank Correlation Coefficients (PRCC). This enables a better understanding of the effects of parameter values on the amplification patterns we observe across our model simulations. In our analysis we include 12 model parameters and assign a uniform probability density function (pdf) to each, with minimum and maximum values chosen in line with the ranges explored in previous sections of this paper; the details of which are outlined below and summarised in Table 2.

In Section 3.3 we explored the effects of affording the EGFR and PDGFRA amplified sub-populations various selection advantages and changing the phylogenetic ordering of mutations. Thus, in order to formally study the sensitivity of our model to these factors we first introduce some new parameters. The parameters $v_{E}^{\rho }$ and $v_{P}^{\rho }$, defined such that $\rho_{E}=v_{E}^{\rho }\rho_{N}$ and $\rho_{P}=v_{P}^{\rho }\rho_{N}$, are the proliferative advantages of the *E* and *P* populations over the *N* population, with minimum and maximum values of 1 and 1.5, i.e., no advantage and a 50% proliferation advantage. Similarly, we investigate the sensitivity of the model to invasive advantages afforded to the amplified sub-populations via the parameters $v_{E}^{D}$ and $v_{P}^{D}$, defined analogously by $D_{E}=v_{E}^{D}D_{N}$ and $D_{P}=v_{P}^{D}D_{N}$. To investigate how the phylogenetic ordering of mutations influences the amplification pattern we see across our simulations in Section 3.3.2, we chose to introduce the EGFR and PDGFRA amplified sub-populations at various introduction times, $t_{E}^{*}$ and $t_{P}^{*}$ determined by the size, *N*_*E*_ or *N*_*P*_, of the growing tumour, which we also include in the sensitivity analysis with minimum and maximum values of 3*N*_*I*_ and 9*N*_*I*_, where *N*_*I*_ is size of the initial population of non-amplified tumour cells.

In addition to these parameters, we include the introduction locations of the mutated populations, $x_{E}^{*}$ and $x_{P}^{*}$, and the proliferation rate, *ρ*_*N*_, and diffusion coefficient, *D*_*N*_, of the non-amplified population of tumour cells (*N*) in our sensitivity analysis. As detailed in Section 3.3, we previously ran simulations for four (*ρ*_*N*_, *D*_*N*_) pairs to represent the heterogeneity of tumours present in our patient cohort and calculated the average proportions from these four simulations, while other parameters in the model were varied. Therefore, we assign each parameter uniform pdfs with minimum and maximum values of 3 and 30, with appropriate units, to represent tumours with varying degrees of proliferative and invasive capabilities.

The interactions, *α*_*PE*_ and *α*_*EP*_, are the final parameters to be included in the sensitivity analysis. We note that we previously made the assumption that the interactions between the EGFR and PDGFRA amplified sub-populations were symmetric, that is *α* = *α*_*PE*_ = *α*_*EP*_. This assumption allowed us to reduce the number of parameters in our model and study the amplification patterns for the three key cases of competition (*α* = −5), cooperation (*α* = 5) and neutralism (*α* = 0) throughout Section 3.3. It is, however, possible that these interactions are non-symmetric and one of the other six interaction types detailed in Table 1 could explain the amplification patterns observed in the biopsy data in Figure 2, or perhaps an interaction scenario where both populations are competing, but not to the same degree. Thus, in the following sensitivity analysis we allow *α*_*PE*_ and *α*_*EP*_ to take different values and sample them independently from uniform distributions with minima and maxima of −5 and 5.

Briefly, the first step of the sensitivity analysis is to conduct the LHS for which we choose a sample size of 2000; each of the 12 parameter distributions given in Table 2 are divided into 2000 intervals of equal probability and a sample is drawn from each. These samples are then randomly grouped and 2000 simulations are run, from which we record the four outputs of interest: the proportions of neither, only EGFR, only PDGFRA and both genes amplified. The inputs and outputs are then rank transformed and PRCC values are calculated between each parameter and each output of interest, with values ranging from −1 to +1. The sign and magnitude of the PRCC values indicate the qualitative relationship between the input and output variables and the importance of parameter uncertainties in accurate prediction of the model outputs [39]. A significance test is conducted to test whether each PRCC value is different from zero, thus indicating any significant correlations between outputs and parameters. A more in depth description of the LHS-PRCC protocol is provided in [39] and [41]. We implemented the sensitivity analysis utilising code by Massey et al. [42], which can be found at https://github.com/scmassey/model-sensitivity-analysis along with further details of the LHS-PRCC method.

**Figure 7.**
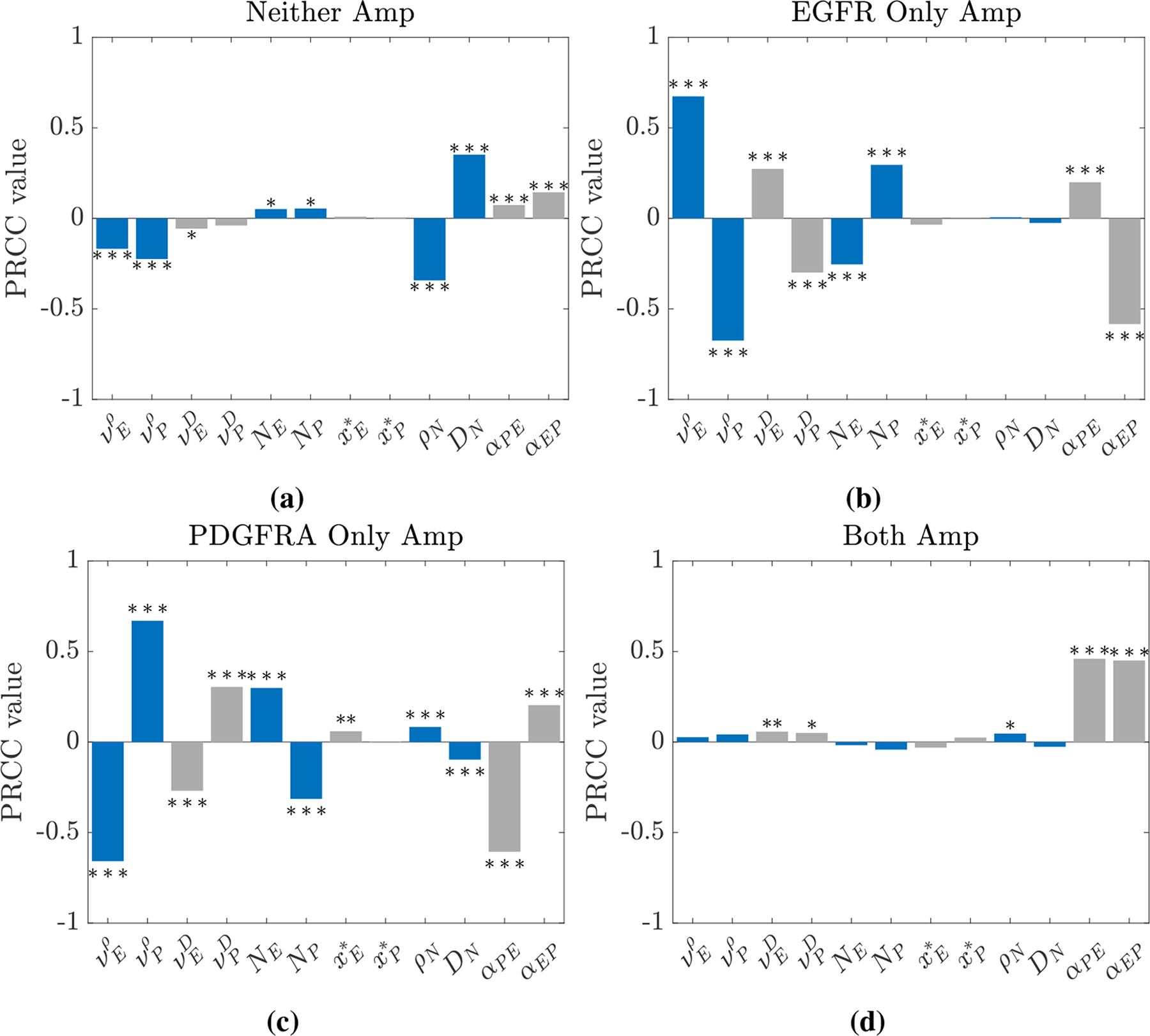
Bar plots showing PRCC values between each model parameter detailed in Table 2 and the four outputs of interest: the proportion of simulations with (a) neither, (b) only EGFR, (c) only PDGFRA and (d) both genes amplified. PRCC values significantly different from zero at the 0.05 (*), the 0.01 (**) and the 0.001 (***) levels are highlighted.

The results from the LHS-PRCC sensitivity analysis for the four outputs of interest from our model, given by Eqs (2.1)–(2.12), and the 12 parameters detailed in Table 2 are shown in Figure 7. As expected from our analysis in Sections 3.3.1 and 3.3.2, the proportions of simulations with only EGFR and only PDGFRA amplified are both strongly correlated to selection advantages and the timings of mutations, with the effects being reflected for each output. For example, there is a strong positive correlation between the parameter $v_{E}^{\rho }$, which affords the EGFR amplified population a proliferative advantage, and the proportion of simulations with only EGFR amplified, whereas there is a strong negative correlation between this parameter and the proportion with only PDGFRA amplified. Affording these amplified populations invasive advantages, through $v_{E}^{D}$ and $v_{P}^{D}$, and introducing them at earlier times, ${\tilde{t}}_{E}$ and ${\tilde{t}}_{P}$, in the growing tumour also has a similar, albeit slightly weaker, effect on the proportions of simulations with only one of the genes amplified observed.

In Figure 7a, we observe that the proportion of simulations with neither gene amplified is most sensitive to the parameters *ρ*_*N*_ and *D*_*N*_. This may be due to the effects at the edges of the simulated tumours, where the total tumour cell population, *T* = *N* + *E* + *P*, is above the threshold of detection and the amplified populations, *E* and *P*, remain undetected below the threshold (as illustrated in Figure 3a), so these points in the simulations contribute to the proportion of the simulation with neither gene amplified. As the parameter *ρ*_*N*_ decreases and *D*_*N*_ increases, the profile of the tumour edges becomes flatter and this area at the edge of the tumour becomes wider, thus contributing more to the proportion of the simulated tumour with neither gene amplified. This suggests that to represent amplification patterns for a population of heterogeneous GBMs it is important to incorporate this heterogeneity into our mathematical modelling by considering a range of *ρ*_*N*_ and *D*_*N*_ values, as we did throughout Section 3.3.

In Figure 7d, we notice that only the interaction parameters, *α*_*PE*_ and *α*_*EP*_, have a strong impact on the proportion of simulations with both genes amplified; this is, again, consistent with our previous observations in Section 3.3, where we saw that the proportion of simulations with both genes amplified increased as we moved from the competitive case through to the neutral and cooperative cases. The other proportions in the simulations are also strongly correlated to these parameters. We observe that if *α*_*PE*_ and *α*_*EP*_ have opposite signs, say *α*_*PE*_ > 0 and *α*_*EP*_ < 0, then the EGFR amplified population will benefit, while the proportion with only PDGFRA amplified will decrease. The case where both parameters have the same sign is more difficult to understand due to the competing effects; a positive *α*_*PE*_ will increase the only EGFR Amp proportion and have a stronger negative effect on the only PDGFRA Amp proportion, whereas a positive *α*_*EP*_, will have the opposite effect. Since the negative effects are stronger in this case, it is likely that the proportions with only one gene amplified will decrease, as the proportion with both amplified will increase when both interaction parameters are positive. Meanwhile, if *α*_*PE*_ and *α*_*EP*_ are both negative, the proportion with both genes amplified will decrease, as the EGFR and PDGFRA amplified sub-populations compete with one another; this is likely to result in each amplified population occupying distinct regions of the tumour or one population dominating, depending on the strength of the competitive interactions and various other factors, such as one population being introduced and becoming established before the other.

Finally, we note that the PRCC values between the introduction locations and each of the four outputs of interest are close to zero, indicating that they are not strongly correlated. While this may be expected, since our results in Figure 6 in Section 3.3.3 and Figures 11 and 12 in the Supplementary Material do not show that changing the introduction locations has a big effect on the amplification patterns observed, it is also possible that this is due to a non-monotonic relationship between the introduction locations and the outputs. In this case, it is possible to remove the non-monotonicity from the problem by dividing the domains of $x_{E}^{*}$ and $x_{P}^{*}$ into two; this process is discussed in further detail in the Supplementary Material, where the results of a second LHS-PRCC analysis with the non-monotonicities accounted for can be found in Figures 13 and 14. Upon removing the non-monotonicity, we find, however, that there are still no very strong correlations between the introduction locations of the amplified sub-populations and the outputs of interest, while the results for the other 10 model parameters remain consistent with the results presented here.

## 4. Discussion

In this paper, we have presented a novel mathematical model of the co-evolution of three distinct tumour cell sub-populations to investigate the nature of interactions between cells with two common mutations occurring in GBMs, namely amplification of the genes encoding the EGFR and PDGFRA proteins. We have used a PDE-based formalism, which reduces to the well known PI model [27–32] if we assume that these genetic differences do not change the phenotype of the cell populations and instead compose a single phenotypically homogeneous population of cancerous cells. We then conducted an *in silico* investigation into the levels of EGFR and PDGFRA amplified sub-populations observed under competitive, cooperative and neutral interactions between these cell types and compared our results to population levels of amplification observed in image-localized biopsy data from a cohort of GBM patients, where a high proportion of biopsies had only the EGFR gene amplified, a smaller proportion had both or neither gene amplified and very few showed amplification of only the PDGFRA gene (see Figure 2b).

In carrying out computational simulations, we found that the amplification patterns observed in simulated tumours under each of the different interaction assumptions did not match those observed across the patient biopsy data when we assumed that cells with the EGFR and PDGFRA genes amplified did not differ in their dynamics, shown in Figure 3b. We note that this was to be expected and is consistent with research suggesting dynamical differences between these sub-populations. For example, a study reconstructing the phylogeny of GBMs suggests that EGFR amplification is a mutation that arises earlier than amplification of the PDGFRA gene [9], while other studies have found that tumours with EGFR amplified are more invasive, which could indicate that EGFR amplified cells themselves are more invasive [14, 15]. Following this, we explored the effects of introducing differences between the sub-populations in our simulations through changing various model parameters. Since a high proportion of biopsies across the patient data have the EGFR gene amplified but no amplification of the PDGFRA gene, we investigated the effects of giving EGFR amplified cells various selection advantages over PDGFRA and non-amplified cell types, shown in Figure 4 and Figure 8 in the supplementary material. While these simulations do not achieve the same amplification patterns observed in the biopsy data, affording EGFR amplified cells a selection advantage does help to produce the high levels of EGFR amplification required, particularly in the competition and neutral interaction cases. We note that further investigation of other degrees of invasive and proliferative advantages may improve the results we see; throughout Section 3.3.1 we only looked at quite large advantages of 50% of the respective parameters, different degrees of advantages are something we may investigate in future work. We then chose to investigate the effects of changing the phylogenetic ordering of mutations and found that introducing the EGFR amplified sub-population earlier in the evolution of our simulated tumours also helped to produce the desired higher level of EGFR amplification under competitive and neutral interaction assumptions, whereas the proportion of points with both genes amplified was much higher than observed in the biopsy data in the cooperative case. Finally, we looked at introducing the EGFR and PDGFRA amplified sub-populations in different locations in the growing tumour simulations. We found that changing these locations did not have such a large effect on the amplification patterns observed as the selection advantages and phylogeny did, however it could be an important factor in replicating amplification patterns observed in individual GBMs with distinct regions of EGFR and PDGFRA amplification. We also conducted a LHS-PRCC sensitivity analysis, which highlighted the sensitivity of the amplification patterns observed across simulations to the selective advantages and introduction times of the amplified sub-populations and the type of interactions occurring between them. Consistent with our finding in Section 3.3.3, this analysis also highlighted that the amplification patterns were not strongly correlated to the introduction locations; while this may be the case, the weak correlation observed could be a result of only considering a small range of tumour locations as a result of the small size of the tumour at early introduction times providing little scope for spatial variation. This is a factor that could be investigated in future work.

Although the simulation results presented in this paper do not perfectly match the patterns of EGFR and PDGFRA amplification observed across the patient biopsy data, our *in silico* modelling approach has allowed us to investigate how different interaction assumptions influence the amplification patterns in simulated tumours and explore the effects of changing the parameters and the timing and position of sub-population introductions in our model; we found that some of these changes improved our simulation results with respect to the biopsy data and were also consistent with suggestions about EGFR amplified sub-populations found in the literature.

In Section 3.3, we only explored each factor individually, whereas a combination of factors relating to the selection advantages of EGFR and PDGFRA amplified cells as well as the timing and location of mutations are likely to be influencing the amplification patterns across the biopsy data. This combination of factors makes it difficult, at this stage, to deduce the nature of interactions between cell types that is driving these patterns. Across our simulation results, however, we did find that the competitive and neutral cases approximated the patterns observed in the biopsy data better than the cooperative case, suggesting that EGFR and PDGFRA amplified sub-populations are not strongly cooperating with one another.

Throughout our investigation in Section 3.3, we only studied one strength of competitive and cooperative interactions between EGFR and PDGFRA amplified sub-populations and also assumed that these interactions were symmetric, whereas these cells may be interacting differently and to stronger or weaker degrees. To shed light on this, in Section 3.4, we allowed *α*_*PE*_ and *α*_*EP*_ to take different values. We found that, if they have opposite signs, one amplified population will benefit while the other decreases and that if they have the same sign but are not equal the results are more difficult to interpret due to competing effects. The effect of this on the amplification patterns observed across a cohort of tumours requires further investigation, which is something we plan to study in future work.

We also note that in this work we have avoided modelling single cell events in a macroscopic setting, by assuming that each of the EGFR and PDGFRA amplified sub-populations only become established within a tumour at most once and introduce a small distribution of cells accordingly. It is possible, however, that multiple mutation events resulting in EGFR and PDGFRA amplification in cells could be occurring in an evolving tumour and it may be more appropriate to model such single cell events in a micro- or meso-scopic setting. However, since we are interested in population level patterns of amplification and are working with large numbers of cells, this would be computationally expensive. In order to better capture single cell events while avoiding a large computational burden, it may be appropriate to consider a hybrid-modelling approach, similar to that described by Smith and Yates [43]. Though in this work, we decided to represent a successful mutation event by introducing a small population of cells in our continuum PDE model and leave such considerations for future work.

Determining the nature of interactions between EGFR and PDGFRA amplified sub-populations in GBMs is a complex biological problem, with factors relating to selection advantages and the phylogeny of these tumours influencing the balance of populations we see in a tumour, as we have demonstrated with our *in silico* investigation in this paper. To be able to untangle the influence from and the nature of interactions from the effects of these other factors, more data may be required. In this study, we had biopsy data for a cohort of 25 patients and a larger number may enable us to gain more insight into the pattern of EGFR and PDGFRA amplification. Furthermore, data at multiple time points, such as biopsies sampled from primary and recurrent tumours, would enable us to extract more dynamic information about the co-evolution of these genetically-distinct sub-populations; this presents challenges, however, as treatment effects would also have to be taken into account. Alternatively, a combination of single- and mixed-cell cultures and mathematical modelling may be able to identify the nature of interactions *in vitro* and provide some useful insights into the co-evolution of EGFR and PDGFRA sub-populations *in vivo* if such data were to become available.

## Supplementary Material

1

## Figures and Tables

**Table 1.** The definitions of interactions that can occur between sub-populations *E* and *P* in our mathematical model as determined by the signs of *α*_*EP*_ and *α*_*PE*_. In this paper we primarily consider the subset of three interactions highlighted in blue.

*α*_*EP*_	*α*_*PE*_	Interaction Type
0	0	Neutralism
< 0	0	Amensalism: *E* negatively affects *P*
0	*<* 0	Amensalism: *P* negatively affects *E*
< 0	< 0	Competition
> 0	0	Commensalism: *E* positively affects *P*
0	> 0	Commensalism: *P* positively affects *E*
> 0	> 0	Cooperation
> 0	< 0	Parasitism: of *P* on *E*
< 0	> 0	Parasitism: of *E* on *P*

**Table 2.** Parameter definitions and the minimum and maximum values of their corresponding uniform distributions.

Parameter	Definition	Min	Max	Units
$v_{E}^{\rho }$	proliferative advantage of *E* cells	1	1.5	unitless
$v_{P}^{\rho }$	proliferative advantage of *P* cells	1	1.5	unitless
$v_{E}^{D}$	invasive advantage of *E* cells	1	1.5	unitless
$v_{P}^{D}$	invasive advantage of *P* cells	1	1.5	unitless
*N*_*E*_	size of tumour when *E* is introduced	3*N*_*I*_	9*N*_*I*_	cells/mm^2^
*N*_*P*_	size of tumour when *P* is introduced	3*N*_*I*_	9*N*_*I*_	cells/mm^2^
$x_{E}^{*}$	introduction location of population *E*	$x_{c}^{*}-1.5$	$x_{c}^{*}+1.5$	mm
$x_{P}^{*}$	introduction location of population *P*	$x_{c}^{*}-1.5$	$x_{c}^{*}+1.5$	mm
*ρ*_*N*_	proliferation rate of *N* cells	3	30	1/year
*D*_*N*_	diffusion coefficient of *N* cells	3	30	mm^2^/year
*α*_*PE*_	effect of *P* on *E*	−5	5	unitless
*α*_*EP*_	effect of *E* on *P*	−5	5	unitless
