# Supplementary material for: Identifying the spatial and temporal dynamics of molecularly-distinct glioblastoma sub-populations
Source: Math Biosci Eng. Author manuscript; Available in PMC 2021 Aug 23. (PMC8382158; doi:10.3934/mbe.2020267)
Supplement: 1 [file NIHMS1720433-supplement-1.pdf]

Supplementary

Here we present additional figures exploring the effects of various selection advantages and the timing and positioning of EGFR and PDGFRA amplified sub-population introductions on the amplification patterns we see in our simulated tumours. These figures show the general trends in changes to the proportions of simulations with neither gene, only the EGFR gene, only the PDGFRA gene and both genes amplified that changing each of these factors produces. We note that the effects of changing each of these factors are symmetric with respect to the proportions of simulations with only EGFR and only PDGFRA amplified. For example, affording E cells a 50% proliferative advantage and P cells no advantages, produces the same simulation proportions of neither and both amplified cells as giving the P population this advantage and E no advantage, while the proportions with only one gene amplified are reflected.

All of the following figures (Figures 8–12) are produced from simulations with the same parameters and assumptions outlined in Section 3.3, apart from where parameter differences are indicated in the figure captions.

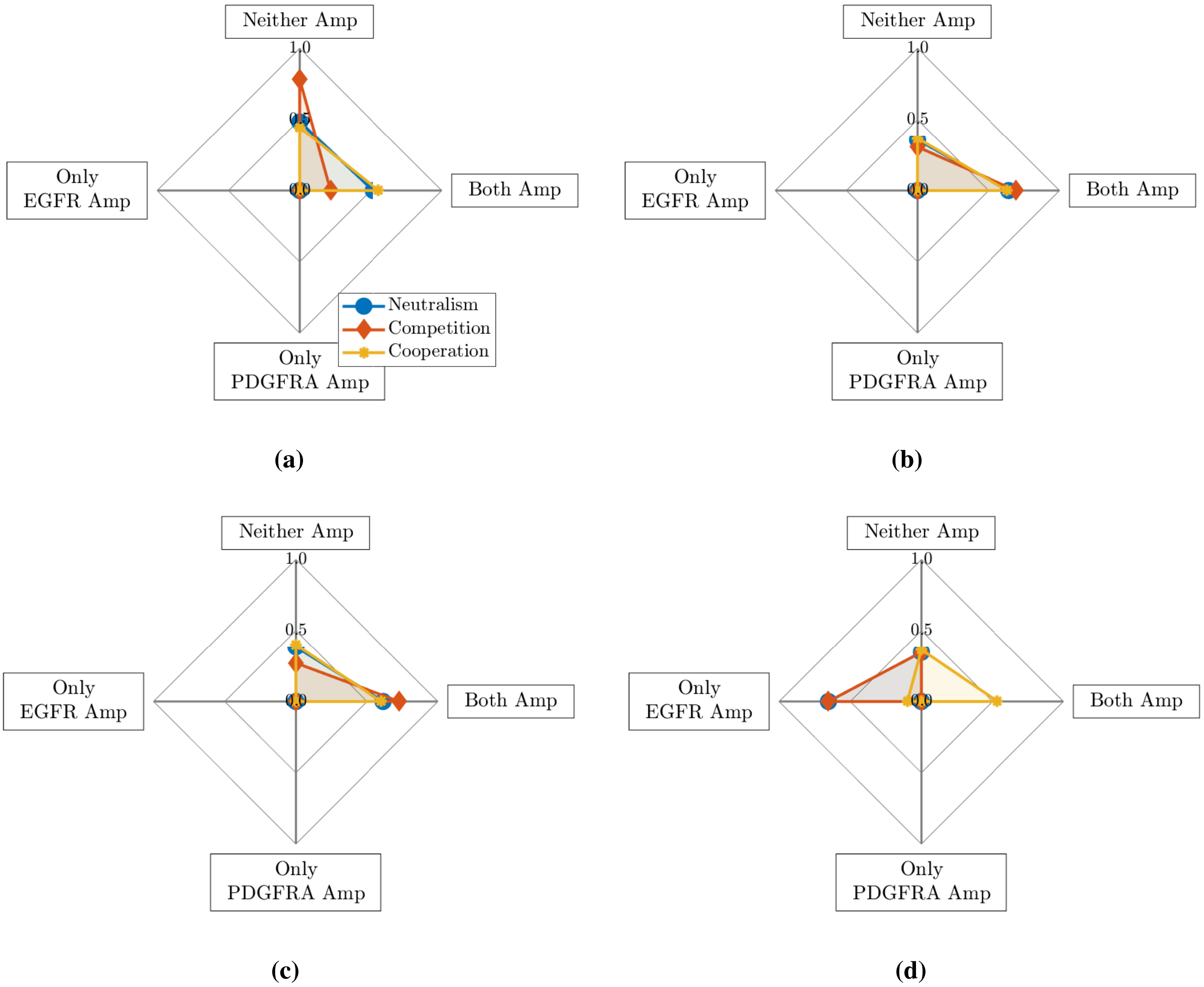

Figure 8. Amplification patterns change when EGFR and PDGFRA amplified sub-populations are afforded proliferative and invasive advantages; proliferative advantages have the bigger impact, decreasing the proportion of the tumour with neither gene amplified. Plot showing the mean proportions of simulations with neither gene (Neither Amp), only the EGFR gene (Only EGFR Amp), only the PDGFRA gene (Only PDGFRA Amp) and both genes (Both Amp) amplified under different interactions when the E and P sub-populations are given various selection advantages: (a) EGFR 50% invasive advantage, PDGFRA 50% invasive advantage ($\rho_E = \rho_N$, $\rho_P = \rho_N$, $D_E = 1.5D_N$ and $D_P = 1.5D_N$); (b) EGFR 50% proliferative advantage, PDGFRA 50% proliferative advantage ($\rho_E = 1.5\rho_N$, $\rho_P = 1.5\rho_N$, $D_E = D_N$ and $D_P = D_N$); (c) EGFR 50% proliferative and invasive advantage, PDGFRA 50% proliferative and invasive advantage ($\rho_E = 1.5\rho_N$, $\rho_P = 1.5\rho_N$, $D_E = 1.5D_N$ and $D_P = 1.5D_N$); (d) EGFR 50% proliferative advantage, PDGFRA 50% invasive advantage ($\rho_E = 1.5\rho_N$, $\rho_P = \rho_N$, $D_E = D_N$ and $D_P = 1.5D_N$).

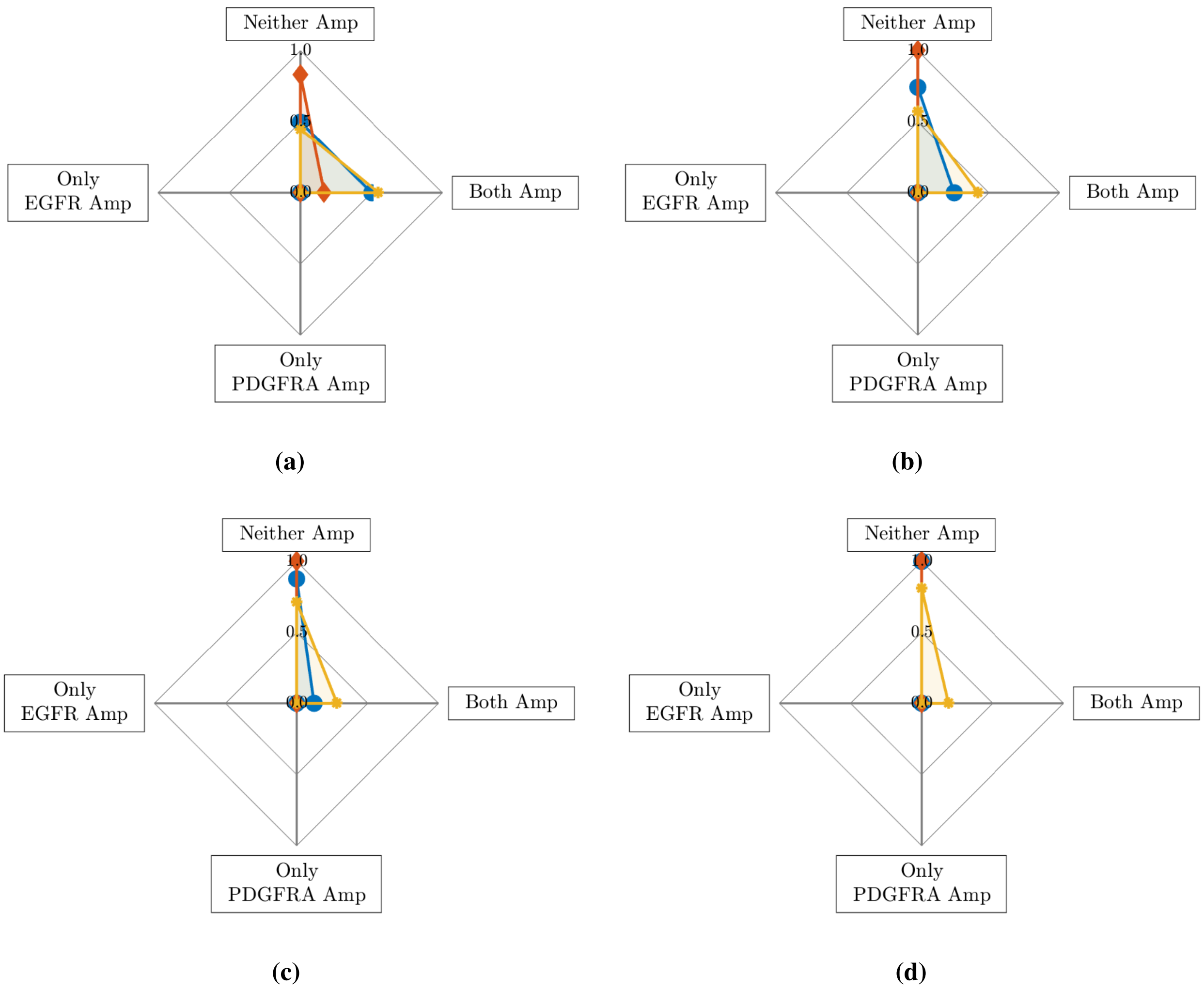

Figure 9. Delaying the introduction of amplified sub-populations increases the proportion of the tumour with neither gene amplified and decreases the amplified proportion. Plot showing the mean proportions of simulations with neither gene (Neither Amp), only the EGFR gene (Only EGFR Amp), only the PDGFRA gene (Only PDGFRA Amp) and both genes (Both Amp) amplified under different interactions when the E and P sub-populations are introduced at the same time which changes: (a) $t_E^* = t_P^* = t_1^*$; (b) $t_E^* = t_P^* = t_3^*$; (c) $t_E^* = t_P^* = t_5^*$; (d) $t_E^* = t_P^* = t_7^*$, as defined in Section 3.3.2.

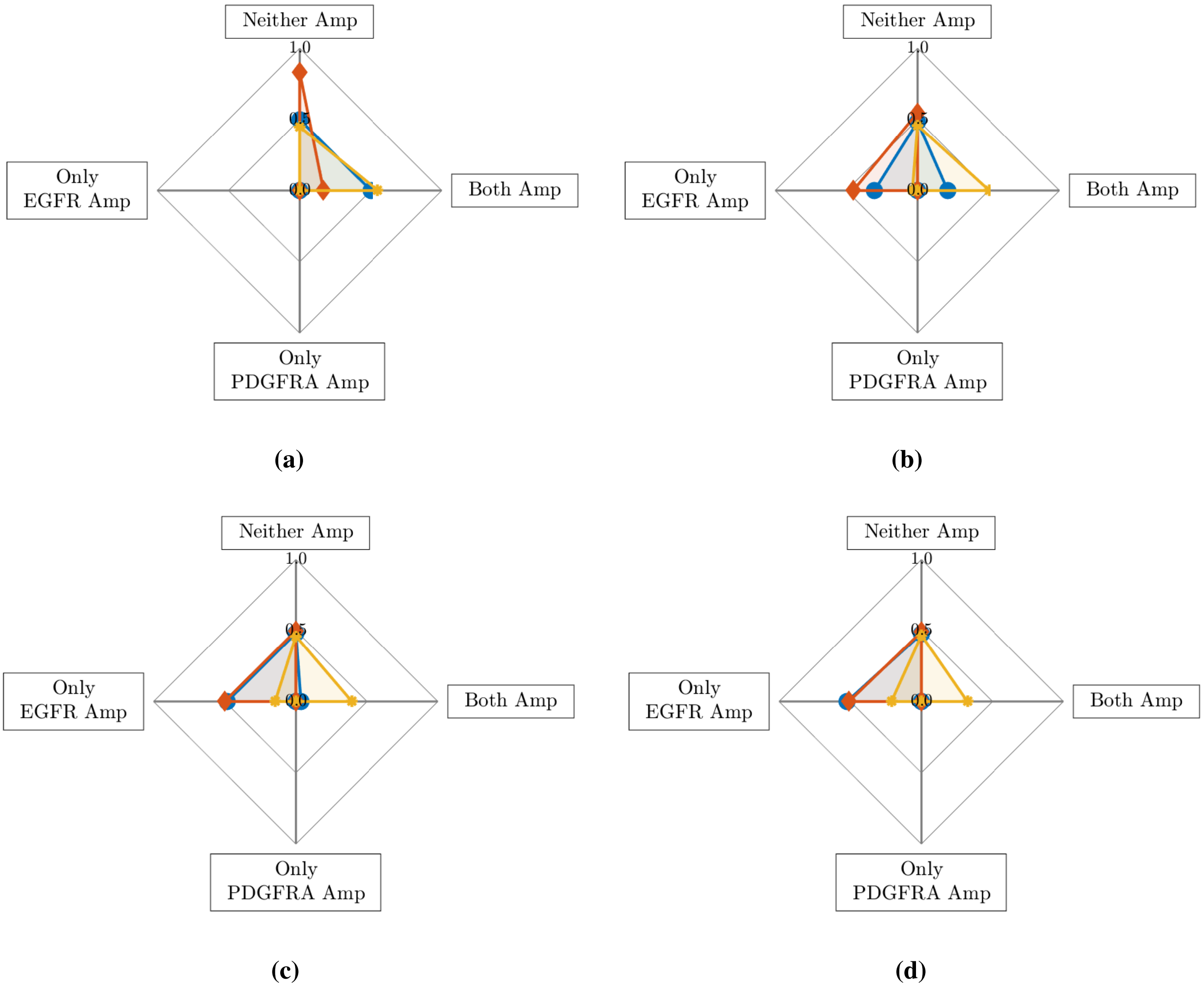

Figure 10. Delaying the introduction of the PDGFRA amplified sub-population increases the proportion of tumour with only EGFR amplified and decreases the both amplified proportion. Plot showing the mean proportions of simulations with neither gene (Neither Amp), only the EGFR gene (Only EGFR Amp), only the PDGFRA gene (Only PDGFRA Amp) and both genes (Both Amp) amplified under different interactions when the E population is introduced at $t_E^* = t_1^*$ and P is introduced at: (a) $t_P^* = t_1^*$; (b) $t_P^* = t_3^*$; (c) $t_P^* = t_5^*$; (d) $t_P^* = t_7^*$, as defined in Section 3.3.2.

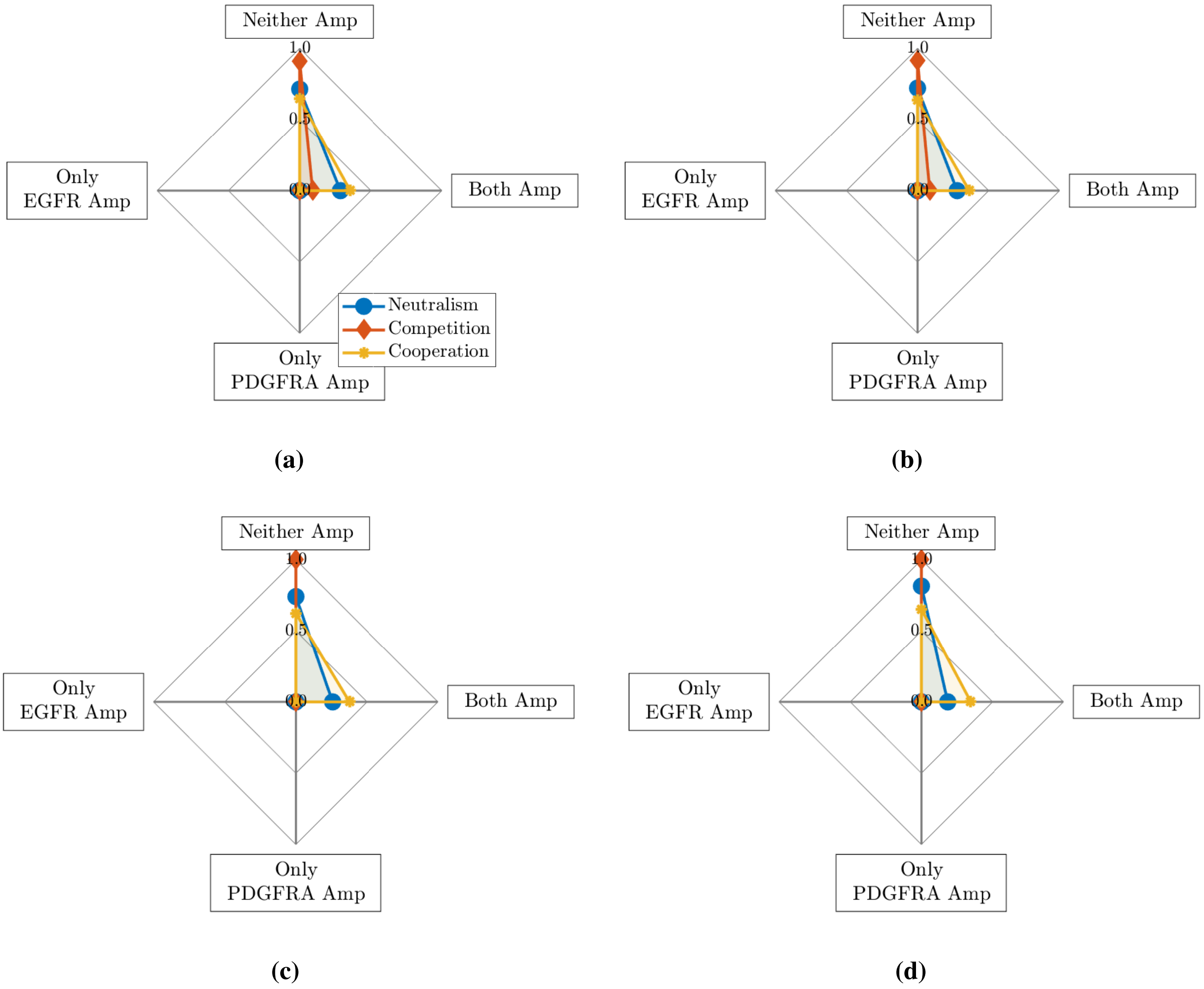

Figure 11. Introducing amplified populations closer to the tumour centre decreases the proportion of tumour with both genes amplified in the neutral and competitive cases, although the effect is small. Plot showing the mean proportions of simulations with neither gene (Neither Amp), only the EGFR gene (Only EGFR Amp), only the PDGFRA gene (Only PDGFRA Amp) and both genes (Both Amp) amplified under different interactions when the E and P populations are introduced at the same location, which changes: (a) $x_E^* = x_P^* = x_1^*$; (b) $x_E^* = x_P^* = x_2^*$; (c) $x_E^* = x_P^* = x_3^*$; (d) $x_E^* = x_P^* = x_4^*$, as defined in Section 3.3.3.

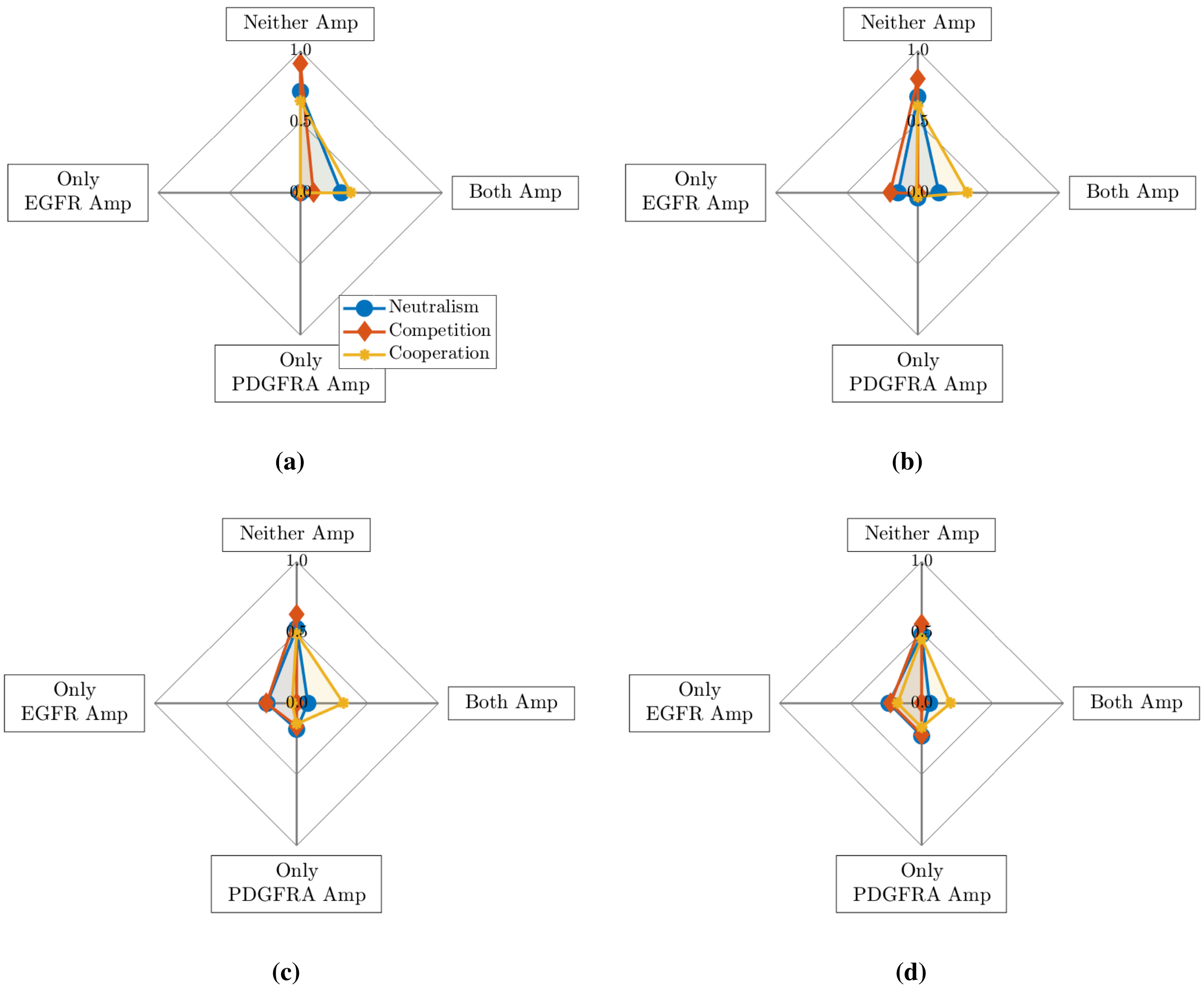

Figure 12. Introducing PDGFRA amplified cells further from the EGFR amplified population decreases the proportion of tumour with both genes amplified and increases the proportions with only one gene amplified. Plot showing the mean proportions of simulations with neither gene (Neither Amp), only the EGFR gene (Only EGFR Amp), only the PDGFRA gene (Only PDGFRA Amp) and both genes (Both Amp) amplified under different interactions when the E population is introduced at $x_E^* = x_1^*$ and P is introduced at: (a) $x_P^* = x_1^*$; (b) $x_P^* = x_3^*$; (c) $x_P^* = x_5^*$; (d) $x_P^* = x_7^*$, as defined in Section 3.3.3.

Effect of Non-monotonicity of Introduction Locations in the LHS-PRCC Sensitivity Analysis

PRCC values provide a measure of the degree of monotonicity between an input and output variable and, therefore, are a good measure of sensitivity for inputs and outputs with monotonic relationships [39, 41]. As Marino et al. [41] demonstrate, a LHS-PRCC sensitivity analysis is not always accurate for input parameters and outputs with non-monotonic relationships and should be treated with caution. We observe that, due to the symmetry of our initial condition $N(x, 0)$, given by Eq (2.4), and, thus, the growing tumour, the relationship between the introduction locations and the proportions observed in our simulations in non-monotonic. For example, in Figure 11 choosing introduction locations $x_E^* =$

$x_P^* = x_5^*$, x_6^* and x_7^* (as defined in Section 3.3.3) will produce equivalent results to those seen when $x_E^* = x_P^* = x_3^*$, x_2^* and x_1^*, respectively, thus non-montonically affecting the proportions of simulations with neither gene, only the EGFR gene and only the PDGFRA gene amplified. Thus, we divide the x_E^* and x_P^* domains into two, over which the relationships are monotonic and conduct a sensitivity analysis in each instance. First we study the case where both E and P are introduced on the right side of the growing tumour and we choose the uniform pdf to have minimum and maximum values of x_c^* and $x_c^* + 1.5$ mm. We note that if we were to instead choose both introduction locations on the left side of the tumour, this would produce analogous results due to symmetry. Secondly, we conduct a sensitivity analysis where E and P are introduced on opposite sides of the tumour; x_E^* is selected from the right side of the tumour and x_P^* from the left. Thus, the pdf for x_E^* remains the same, but x_P^* is instead assigned a uniform distribution with minimum and maximum values of $x_c^* - 1.5$ and x_c^* mm, respectively. All other parameters and their distributions are kept the same as detailed in Table 2. The results from these two LHS-PRCC sensitivity analyses are shown in Figures 13 and 14. In Figure 13, we see that the PRCCs between the introduction location parameters and each of the outputs of interest do not show a strong correlation when both x_E^* and x_P^* are selected from the same side of the growing tumour. However, in Figure 14, a weak, but significant, correlation is present between the location parameters and the proportion of simulations with only the EGFR or only the PDGFRA gene amplified when x_E^* and x_P^* are selected from opposite sides of the growing tumour, whereas there are no correlations with the other two outputs of interest. Importantly, the results for the 10 other model parameters are consistent with the main LHS-PRCC analysis presented in Section 3.4.

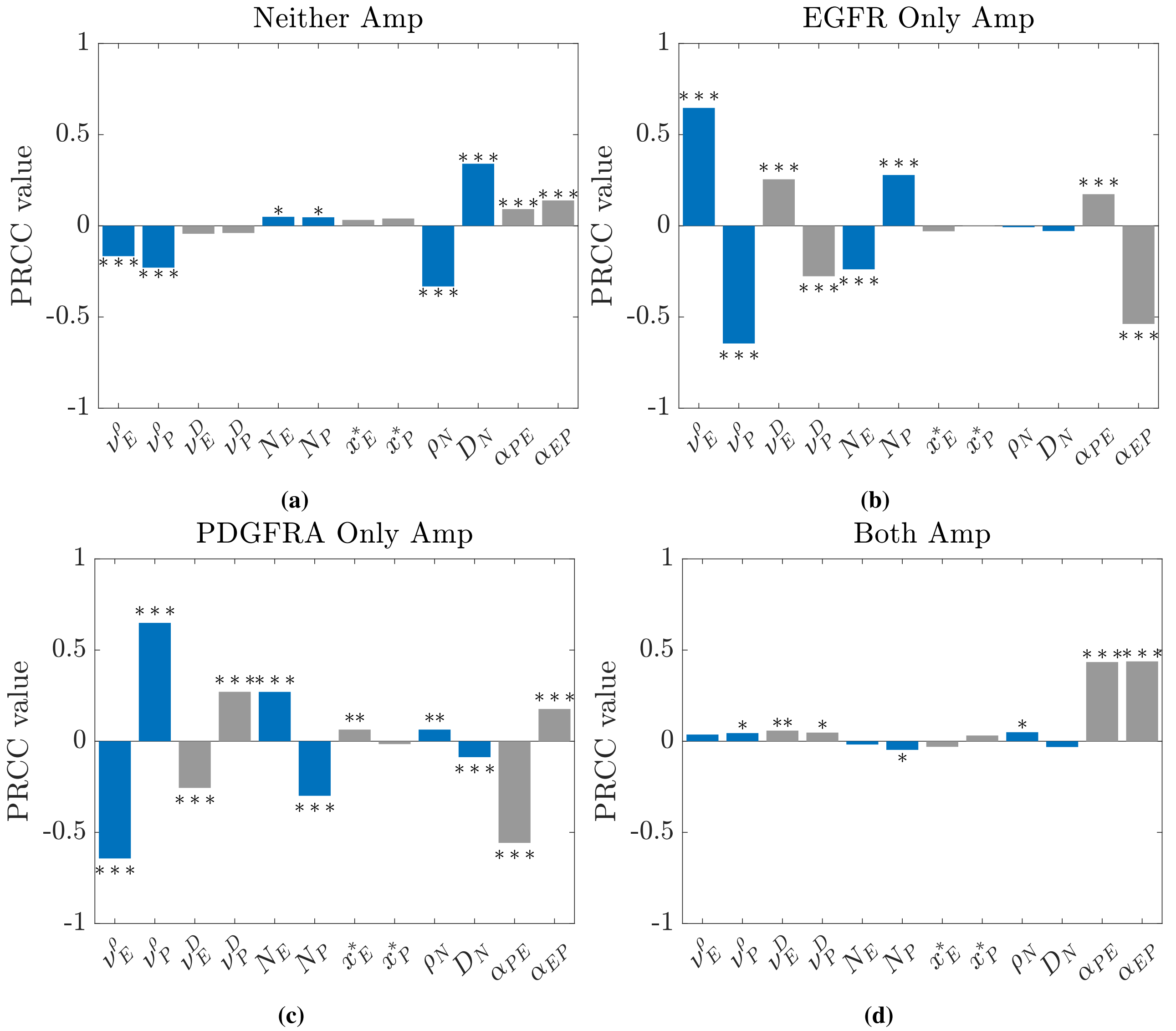

Figure 13. Sensitivity analysis for tumour composition when both mutations arise on the same side of the tumour. Bar plots showing PRCC values between each unknown model parameter and the four outputs of interest: the proportion of simulations with (a) neither, (b) only EGFR, (c) only PDGFRA and (d) both genes amplified. All samples for the LHS step are drawn from the parameter distributions in Table 2, apart from the locations parameters x_E^* and x_P^* which are both drawn from a uniform distribution with minimum and maximum values of x_c^* and $x_c^* + 1.5$mm. Significant results at the 0.05 (*), the 0.01 (**) and the 0.001 (***) levels are highlighted.

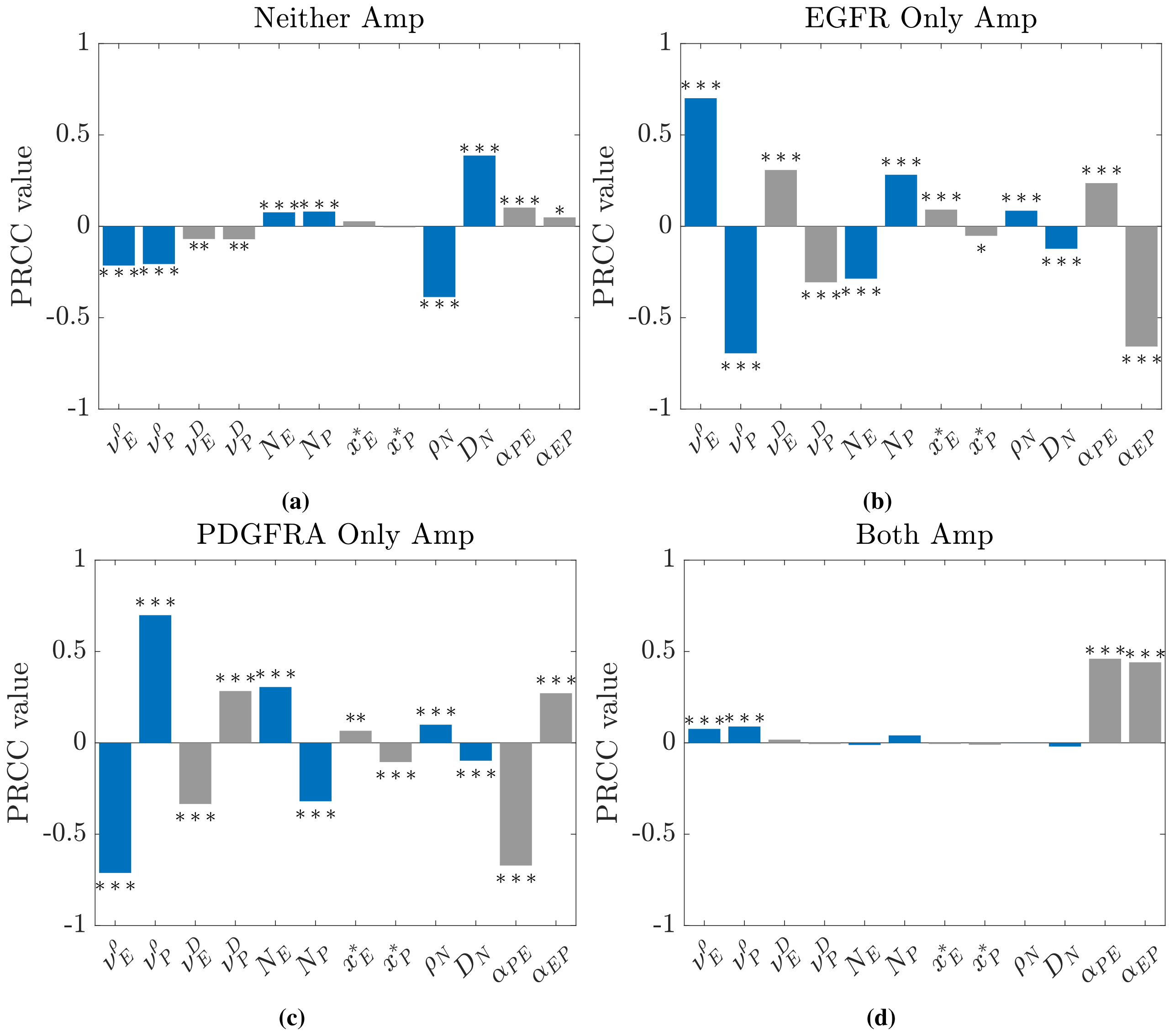

Figure 14. Sensitivity analysis for tumour composition when the two mutations arise on opposite side of the tumour. Bar plots showing PRCC values between each unknown model parameter and the four outputs of interest: the proportion of simulations with (a) neither, (b) only EGFR, (c) only PDGFRA and (d) both genes amplified. All samples for the LHS step are drawn from the parameter distributions in Table 2, apart from the locations parameters x_E^* and x_P^*; x_E^* is drawn from a uniform distribution with minimum and maximum values of x_c^* and $x_c^* + 1.5$mm; x_P^* is drawn from a uniform distribution with minimum and maximum values of $x_c^* - 1.5$ and x_c^*mm. Significant results at the 0.05 (*), the 0.01 (**) and the 0.001 (***) levels are highlighted.
